# Retinoblastoma vulnerability to combined *de novo* and salvage pyrimidine ribonucleotide synthesis pharmacologic blockage

**DOI:** 10.1016/j.heliyon.2023.e23831

**Published:** 2023-12-17

**Authors:** Tanzina Mollick, Suhas Darekar, Basile Dalarun, Flavia Plastino, Juan Zhang, Andres Pastor Fernández, Twana Alkasalias, Helder André, Sonia Laín

**Affiliations:** a Department of Microbiology, Tumor and Cell Biology, Karolinska Institutet, 17165, Solna, Stockholm, Sweden; b Department of Clinical Neuroscience, Division of Eye and Vision, St. Erik Eye Hospital, Karolinska Institutet, 17177, Stockholm, Sweden; c General Directorate of Scientific Research Center, Salahaddin University-Erbil, Erbil, Kurdistan Region, Iraq

**Keywords:** Dhodh, Uridine uptake, Apoptosis, Cell cycle, Genome alteration

## Abstract

Retinoblastoma is an eye cancer that commonly affects young children. Despite significant advances, current treatments cause side effects even when administered locally, and patients may still have to undergo enucleation. This is particularly disheartening in cases of bilateral retinoblastoma. Hence, there is an urgent need for novel therapeutic strategies. Inhibitors of the enzyme dihydroorotate dehydrogenase (DHODH), which is involved in the *de novo* pyrimidine ribonucleotide synthesis pathway, have proven to be effective in preclinical trials against several cancers including pediatric cancers. Here we tested whether blocking pyrimidine ribonucleotide synthesis promotes retinoblastoma cell death. Cultured retinoblastoma cell lines were treated with small molecule inhibitors of DHODH alone or in combination with inhibitors of nucleoside uptake to also block the salvage pathway for pyrimidine ribonucleotide formation. On their own, DHODH inhibitors had a moderate killing effect. However, the combination with nucleoside uptake inhibitors greatly enhanced the effect of DHODH inhibition. In addition, we observed that pyrimidine ribonucleotide synthesis blockage can cause cell death in a p53 mutant retinoblastoma cell line derived from a patient with metastasis. Explaining these results, the analysis of a published patient cohort revealed that loss of chr16q22.2 (containing the DHODH gene) is amongst the most frequent alterations in retinoblastoma and that these tumors often show gains in chromosome regions expressing pyrimidine ribonucleotide salvage factors. Furthermore, these genome alterations associate with malignancy. These results indicate that targeting pyrimidine ribonucleotide synthesis may be an effective therapeutic strategy to consider as a treatment for retinoblastoma.

## 1 Introduction

Retinoblastoma is the most common primary eye cancer in children with a frequency of 1 per 15,000 births worldwide. Retinoblastoma typically occurs in children younger than 5 years and accounts for 2 % of all pediatric cancers. Retinoblastoma usually begins in the developing retina with the inactivation of the *RB1* tumor suppressor gene, followed by further genomic abnormalities. Hence, this cancer most frequently results from the loss of the major G1-S cell cycle checkpoint regulator protein Rb [1]. Although mutations in *RB1* are often considered necessary for retinoblastoma initiation, the transition into retinoblastoma may require further changes in key genes, such as *KIF14, E2F3, MYCN, DDX1, MDM4, OTX2, DEK, CDH1, BCOR and RBL2* [2].

Current treatment regimens cause side effects even when administered locally to the eye. These include vitreous seeding, retinal tears, retinopathy, retinal pigment epithelium damage and partial to full loss of vision [[3], [4], [5]], and patients may still have to undergo enucleation [6]. Nevertheless, patient survival rate for retinoblastoma has increased from 5 % to more than 95 % over the past decade and enucleation is avoided in 95 % of the cases of advanced unilateral disease [6]. This is in part due to the ophthalmic artery technique (intraarterial delivery of chemotherapy to the ostium of the ophthalmic artery) and the use of DNA alkylators such as melphalan. Unfortunately, melphalan can be toxic to the retina in as little as a single injected dose. Indeed, melphalan alkylates guanine and causes covalent linkages between strands of DNA. This inhibits DNA and RNA synthesis, causing toxicity in both dividing and non-dividing tumor and normal cells. Furthermore, these effects on DNA may lead to further genomic instability in the tumor cells and the acquisition of genomic alterations by normal cells. The frequency of metastatic retinoblastoma ranges from 4.8 to 11 %. Common sites of extraocular retinoblastoma include the orbit, preauricular nodes, bones, central nervous system and liver. These patients need to be treated systemically using high doses of DNA damaging agents that can cause second cancers later in life, especially if the patients bear germ line mutations as retinoblastoma patients often do. Altogether, this argues for an urgent need in identifying new treatments that are less toxic to normal cells than melphalan and other DNA damaging agents such as carboplatin.

Targeting pyrimidine ribonucleotide synthesis, specifically by inhibiting the enzyme dihydroorotate dehydrogenase (DHODH), has recently shown promising preclinical results in different types of cancers including pediatric cancers [[7], [8], [9], [10]]. DHODH inhibition in several cancer cell lines initially leads to an S phase accumulation, which is later followed by a cell death response [11]. Moreover, DHODH inhibition can activate the tumor suppressor function of p53 [11,12]. Since p53 in retinoblastoma is typically wild-type and the G1-S checkpoint regulator Rb is altered, we envisaged targeting pyrimidine ribonucleotide synthesis through DHODH inhibition as a viable option to accumulate cells in S phase and induce a cell death response.

Pyrimidine ribonucleotides are synthesized through *de novo* and salvage pathways. For a scheme of these pathways see Ref. [10]. In the *de novo* pathway, DHODH, which is localized on the inner mitochondrial membrane, catalyzes the oxidation of dihydroorotate to orotate by using ubiquinone as an electron acceptor [13]. The central product of the pathway and a precursor to all pyrimidine ribonucleotides is uridine monophosphate (UMP). UMP can be synthesized also by the salvage pathway through the cellular uptake of uridine, recycling of nucleic acids or conversion of cytidine to uridine by cytidine deaminases [13]. The cellular uptake of uridine relies on the activity of human equilibrative nucleoside transporters hENT1-3 (encoded by *SLC29A1-3*) and concentrative nucleoside transporters (hCNT1-3 encoded by *SLC28A1-3*) [14]. The relative contribution of the *de novo* or salvage pathways may vary between cell types. It has been suggested that highly proliferative cancer cells may be more dependent on the *de novo* pathway, whereas slow growing, resting cells and fully differentiated cells opt for the less energy demanding salvage pathway [15,16]. Although DHODH has been considered as an attractive target for cancer therapy, the efficacy of DHODH inhibitors is bound to be debilitated by physiological uridine levels. Thus, a combination approach to block both the *de novo* and salvage pathways of pyrimidine ribonucleotide synthesis could be a reasonable strategy to consider for cancer therapy. Indeed, such a combination has been proven effective in cell culture and in *vivo* cancer models [[17], [18], [19], [20]].

In this study we tested whether targeting pyrimidine ribonucleotide synthesis would be a suitable option for retinoblastoma therapy. A major challenge in this work was that most retinoblastoma cell lines are difficult to grow in culture. Furthermore, those that do proliferate in culture do so slowly. Hence, despite their defect in Rb expression, low levels of cell death upon pyrimidine ribonucleotide synthesis blockage could be expected. As shown here, this was the case when DHODH inhibitors (DHODHi) were used as single agents. However, administering DHODHi with nucleoside uptake inhibitors to also block the salvage pathway, markedly enhanced the killing effect of DHODH inhibition suggesting that this combination may be a viable approach for retinoblastoma treatment or to sensitize retinoblastoma to current treatments with alkylating agents such as melphalan.

## 2 Materials and Methods

### 2.1 RNA expression analyses

Expression of RB1, RBL2, MYCN and CDA mRNAs in a selection of tumor types was performed using the R2 Genomics Analysis and Visualization Platform (http://r2.amc.nl) as described in the figure legends.

### 2.2 Cell culture

Weri-Rb1 (HTB-169™), Y79 (HTB-18™) and ARPE-19 (CRL-2302™) cell lines were purchased from ATCC. NCC-RbC-51 (RCB2206), NCC-RbC-60 and NCC-RbC-83 were purchased from Riken BioResource Center. ARN8 cells were derived from the A375 melanoma cell line as described previously [21]. Unless otherwise stated, cell lines were grown as follows. The Weri-Rb1 cell line was grown in RPMI-1640 (R8758, Sigma-Aldrich) supplemented with 10 % FBS (SV30160.03, HyClone) and 100 U/mL of pen/strep (SV30010, HyClone). The Y79 cell line was grown in RPMI-1640 supplemented with 20 % FBS and 100 U/mL of pen/strep. The NCC-RbC-51 cell line was grown in RPMI-1640 supplemented with 20 % FBS, 1 mM sodium pyruvate (P5280, Sigma-Aldrich) and 100 U/mL of pen/strep. The NCC-RbC-60 and NCC-RbC-83 cell lines were grown in RPMI-1640 supplemented with 20 % FBS, 50 μM 2-mercaptoethanol (P5280, Sigma-Aldrich) and 100 U/mL of pen/strep. The ARN8 cell line was grown in DMEM (D6429, Sigma-Aldrich) supplemented with 10 % FBS and 100 U/mL of pen/strep and the ARPE-19 cell was grown in DMEM/F12 (D8437, Sigma-Aldrich) supplemented with 10 % FBS and 100 U/mL of pen/strep. Where indicated, FBS was substituted by serum replacement 3 (S2640, Sigma-Aldrich). All cells were maintained in a 95 % air and 5 % CO_2_ humidified incubator at 37 °C. All retinoblastoma cell lines were non-adherent and were grown as suspension cultures. ARPE-19 and ARN8 cell lines were adherent. Cell lines were checked for mycoplasma contamination using the MycoAlert kit (LT07-318, Lonza).

### 2.3 Compounds

Brequinar sodium (SML0113), NBMPR (N2255), uridine (U3003), staurosporine (S5921) and nutlin-3 (N6287) were purchased from Sigma-Aldrich. BAY2402234 was purchased from Med Chem Express (HY-112645). Dipyridamole (DP) was purchased from Selleck Chemicals (S1895). All inhibitors were dissolved in DMSO (D8418, Sigma-Aldrich).

### 2.4 WST-1 assay

Cells were seeded at a density of 25,000 cells/100 mL in 96 well plates. Next day, cells were treated with 10 μL of varying concentrations of compounds or vehicle (DMSO) in triplicate. The characteristic color of DP was accounted for by transferring 10 μL of similar concentrations of DP to wells only containing medium. After 72 h of treatment, 10 μL of WST-1 reagent (11644807001, Sigma-Aldrich) was added to each well. The plate was then transferred to an incubator at 37 °C and 5 % CO_2_ for 2 h. Absorbance values were then measured on a Cytation 5 plate reader (BioTek). In case of DP, absorbance values from wells only containing medium were subtracted from those with cells treated with similar concentrations, to compensate for compound color. Data were plotted using GraphPad Prism 8 software showing mean + SD values for 3 technical replicates. IC50 values for BQ were calculated as the weighted mean + weighted SD as previously shown [22] for 4 biological replicates (n = 4).

### 2.5 Cell cycle and live/dead flow cytometry

Cells were seeded at a density of 50,000 cells/mL in T25 flasks for retinoblastoma cell lines or 6 well plates for the ARPE-19 cell line in the indicated media. The following day, cells were treated with compounds. After the desired treatment times, cells were collected in Falcon tubes, centrifuged and washed in PBS (D8537, Sigma-Aldrich).

In the live/dead assays, after washing with PBS, cells were resuspended in 300 μL of LIVE/DEAD™ fixable green dead cell stain (L23101, Invitrogen) dissolved in PBS at a dilution of 1:1000. After 30 min of incubation in the dark at 4 °C, cells were washed with PBS containing 3 mM of EDTA (E177, Amresco) (PBS/EDTA) and finally resuspended in 1 mL ice-cold PBS/EDTA followed by fixation by adding the suspension to 3 mL of ice-cold absolute ethanol in a dropwise manner while vortexing. Cells were kept at −20 °C for at least 12 h.

For cell cycle analyses, cells were resuspended in 1 mL of ice-cold PBS/EDTA and fixed in ethanol as described above. Fixed cells were washed twice in PBS/EDTA containing 3 % FBS and finally resuspended in 500 μL of PBS/EDTA containing 300 μg/mL propidium iodide (P3566, Invitrogen) and 150 μg/mL RNAse A (12,091–021, Invitrogen). Cells were incubated in this solution for 30 min at room temperature in the dark.

Live/dead and cell cycle analyses were performed on a Becton Dickinson FACScan or FACSCalibur (BD Biosciences) flow cytometer. Data was analyzed using FlowJo software V10.2. Cell cycle and live/dead assay bar graphs were prepared using GraphPad Prism 8 software. In the case of live/dead assay graphs, values are shown as mean + SD for 3 technical replicates.

### 2.6 ^3^H-uridine uptake assay

The procedure is based on described method [11]. ARN8 cells were seeded at a density of 100,000 cells/mL in 6 well plates. The following day, the medium was discarded and the cells were washed twice with transport buffer (20 mM trizma hydrochloride (T3253, Sigma-Aldrich), 130 mM sodium chloride (71,382, Sigma-Aldrich), 3 mM potassium phosphate dibasic trihydrate (P9666, Sigma-Aldrich), 1 mM magnesium chloride (M8266, Sigma-Aldrich), 5 mM D-(+)-glucose (15,023–021, Invitrogen) and 2 mM calcium chloride dihydrate (223,506, Sigma-Aldrich) dissolved in deionized water, pH 7.4). The cells were then treated for 15 min with 0.5 mL of different concentrations of DP or vehicle (DMSO), diluted in transport buffer. Afterwards, cells were treated for 60 s with 250 μL of 12 μCi/mL 3H-uridine (NET367001MC, PerkinElmer) dissolved in transport buffer. The uptake of 3H-uridine was immediately stopped by multiple washes with ice-cold transport buffer supplemented with 1 mM unlabeled competing uridine. Cells were harvested in 10 % sodium dodecyl sulphate (SDS) (71,736, Sigma-Aldrich) and then transferred to a 24 well flexible plate. After adding optiphase supermix (1200–439, PerkinElmer) to the samples, the counts per minute were measured using a 1450 MicroBeta JET liquid scintillation counter (PerkinElmer/Wallac). The mean + SD values for 2 technical replicates were obtained using GraphPad Prism 8 software.

### 2.7 5-Ethynyl uridine uptake assay

Weri-Rb1 cells were seeded at a density of 50,000 cells/mL using RPMI-1640 medium supplemented with serum replacement 3 (S2640, Sigma-Aldrich) and 100 U/mL of pen/strep in T25 flasks. The next day, the cells were treated with 10 μM of DP or vehicle. After 24 h compound incubation, cells were treated with 50 μM of 5-ethynyl uridine (E10345, Invitrogen) for 1 h. Immediately before addition, the cells were gently pipetted up and down to reduce cell clumps. After 5-ethynyl uridine treatment, cells were centrifuged, washed with PBS and then fixed overnight at 4 °C using Fix/Perm buffer solution (eBiosciences). Next, the cells were permeabilized with a saponin based permeabilization buffer (C10632, Invitrogen) for 20 min at 4 °C. The cells were then washed with PBS containing 1 % BSA (A9647, Sigma-Aldrich) and blocked with the same solution for 30 min at room temperature. Afterwards, a click-it reaction was performed with an Alexa Fluor™ 488 Azide (A10266, Invitrogen) using a Click-iT™ cell reaction buffer kit (C10269, Invitrogen) according to the manufacturer's instructions. Cells were washed once with blocking solution, twice with PBS containing 0.1 % Tween 20 (P9416, Sigma-Aldrich) and once with PBS/EDTA. Finally, the cells were resuspended in PBS/EDTA and data acquired on the Amnis ImageStream X mkII imaging flow cytometer. The acquisition was set to 1000 cells or maximum acquisition time of 20 min. Single cells in focus were analyzed using the IDEAS Analysis Software. The mean fluorescence intensity values were plotted as mean + SD for 2 technical replicates using GraphPad Prism 8.

### 2.8 Real time live cell imaging

Weri-Rb1 and Y79 cells were seeded at a density of 150,000 cells/mL and ARPE-19 cells were seeded at a density of 50,000 cells/mL in 96 well plates (100 μL per well). The following day, cells were treated with vehicle (DMSO), compounds as single agents and compounds in combination diluted in medium supplemented with either caspase 3/7 red reagent (4704, Sartorious) or YOYO-3 reagent (Y3606, Invitrogen). The final concentration of caspase reagent added to the cells was 0.5 μM and of YOYO-3 was 50 nM. Prewarmed sterile water was added to the spaces in between the wells to reduce evaporation. The plates were immediately transferred to the IncuCyte S3 system located inside a humidified incubator and 5 % CO_2_ at 37 °C. Images were taken 40 min after compound addition to plates to account for condensation. For the retinoblastoma cell lines, 9 images per well were taken at 20x magnification after every 4 h for a total time period of 6 days. For the ARPE-19 cell line, 5 images per well were taken at 10x magnification after every 4 h for a total time period of 6 days. Image analysis was performed with the IncuCyte S3 2018A Rev 1 software. The caspase 3/7 and YOYO-3 positive cell counts were normalized by measurement of cell confluency %. Normalized data were plotted as mean + SD for 3 technical replicates using GraphPad Prism 8. In case of the ARPE-19 cell line, YOYO-3 data is shown starting from the 8 h time point due to high background in the earlier time points. Adobe Photoshop CC (version 2015.5) was used to adjust the contrast, brightness and sharpness of the ARPE-19 live cell images in Supplementary Fig. 7C, and also to make the inserted magnified images in Supplementary Fig. 7.

### 2.9 Western blotting

To check basal DHODH protein levels in different cell lines, subconfluent cells were harvested, washed in PBS and cell pellets lysed in 1x SDS lysis buffer (32.9 mM Tris-HCl (T5941, Sigma-Aldrich) pH 6.8, 13.5 % glycerol (G5516, Sigma-Aldrich) and 1.05 % SDS). For experiments using compound treatments, Weri-Rb1 and Y79 cells were seeded at a density of 50,000 cells/mL in T25 flasks. The following day, cells were treated as indicated, harvested, washed in PBS and lysed in SDS lysis buffer. After lysis, all samples were heated at 96 °C for 5 min followed by sonication. Protein concentrations were measured using the BioRad DC Protein Assay kit (50–0116, BioRad) according to the manufacturer's protocol. After normalizing protein concentrations, Laemmli sample buffer (161–0737, BioRad) and dithiothreitol (to a final concentration of 100 mM) (D0632, Sigma-Aldrich) were added. Samples were heated at 96 °C for 5 min and loaded in 10-well 4–15 % mini-PROTEAN TGX stain-free gels (4,568,084, BioRad) in Tris-glycine running buffer (161–0732, BioRad). The Precision Plus Protein all blue ladder was used (1,610,373, BioRad). After gel electrophoresis at 160 V, gels were activated for 5 min using the ChemiDoc Touch system (170–8370, BioRad). Proteins were then transferred to PVDF membranes available with the Trans-Blot Turbo kit (170–4150, BioRad) using the Trans-Blot Turbo transfer system from BioRad. Images of the stain free membrane, representing total protein, were taken with the ChemiDoc Touch. Membranes were then blocked in 5 % fat-free milk dissolved in PBS containing 0.1 % Tween 20. All primary antibodies were incubated overnight at 4 °C on a shaker. All secondary antibodies were incubated at room temperature for 1 h on a shaker. Membranes were developed using the Clarity Western ECL Substrate (170–5061, BioRad) and images were taken with ChemiDoc Touch system. All primary antibodies were incubated overnight at 4 °C on a shaker. All secondary antibodies were incubated at room temperature for 1 h on a shaker. Membranes were developed using the Clarity Western ECL Substrate (170–5061, BioRad) and images were taken with ChemiDoc Touch system (170–8370, BioRad). Primary antibodies used were a mixture of clones 1.1 and 3.1 of mouse monoclonal antibodies against DHODH (1:500, 43 kDa) kindly provided by Dr. Marta Nekulova (Masaryk Memorial Cancer Institute, Brno, Czech Republic), mouse monoclonal against p53 DO1 (1:1000, 53 kDa) kindly provided by Dr. Borivoj Vojtesek (Masaryk Memorial Cancer Institute, Brno, Czech Republic), rabbit monoclonal against cleaved PARP-1 Y34 (1:1000, 85 kDa) (ab32561, Abcam), mouse monoclonal against MYCN (sc-53993, Santa Cruz) and mouse monoclonal GAPDH (1:5000, 37 kDa) (ab8245, Abcam). Secondary antibodies were either rabbit anti-mouse HRP (P0261, DAKO) or swine anti-rabbit HRP (0217, DAKO). Analysis of pixel densities of blots were done with the Image Lab 5.2.1 software and later plotted with GraphPad Prism 8.

### 2.10 Statistical analysis

Statistical significance (p values: * <0.05, ** <0.01, *** <0.001, ****<0.0001) was determined using one-way analysis of variance (ANOVA) followed by Dunnette's multiple comparison tests or using Student's t-test with GraphPad Prism 8.

### 2.11 Analysis of genomic alterations

Analysis of genomic alterations related to annotated genes was performed using published data on a retinoblastoma 102 patient cohort [23].

## 3 Results

### 3.1 Shared features of DHODH sensitive cancers and retinoblastoma

**Fig. 1:**
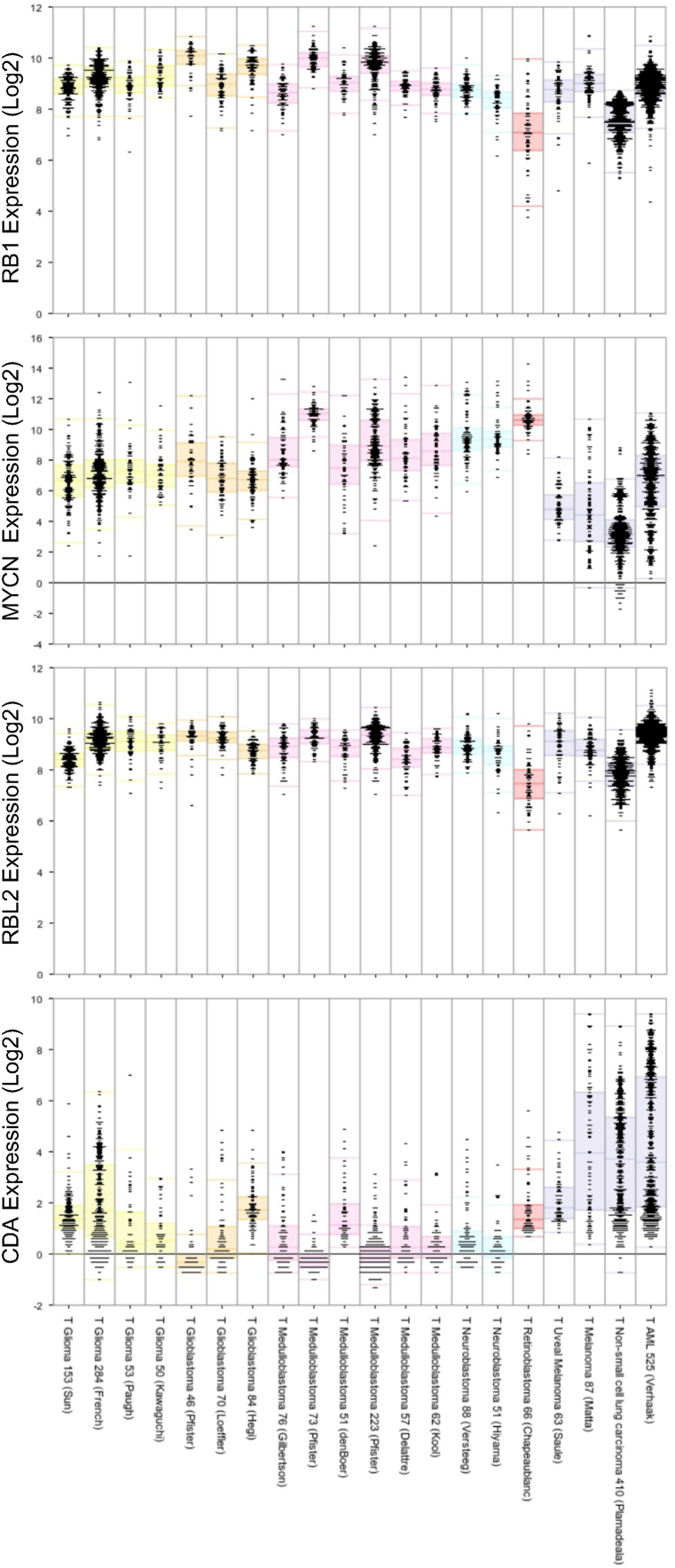
Expression of *RB1*, *RBL2*, *MYCN* and *CDA* in tumors measured as MAS5.0 were obtained using the MegaSampler tool in the R2 Genomics Analysis and Visualization Platform (https://hgserver1.amc.nl/cgi-bin/r2/main.cgi). Number of samples in each study is indicated. Studies on glioma, glioblastoma, medulloblastoma and neuroblastoma containing more than 40 tumor samples are included. The studies involving the highest number of melanoma, non-small lung carcinoma and acute myeloid leukemia samples were chosen for comparison together with the study on uveal melanoma.

Previous work demonstrated that small cell lung cancers (SCLC) are particularly susceptible to DHODH inhibition [24]. Interestingly, from a genetic point of view retinoblastoma shares some features with SCLC. Notably, like retinoblastoma, SCLCs are frequently deficient for *RB1* function [25], hence, in both cancers it is likely that the G1/S checkpoint is defective. In addition, both tumor types may overexpress MYC paralogs [2,25]. By analyzing the levels of expression of transporters and enzymes involved in the synthesis of pyrimidine nucleotides in cell lines by using the depmap portal (https://depmap.org/portal/), in our previous publication we have observed that the expression of the mRNA for cytidine deaminase CDA is low in SCLC cell lines [26]. Weri-Rb1 and Y79 are the only retinoblastoma cell lines analyzed in the depmap database, and both express low levels of CDA mRNA. As shown by analyzing the data in the R2 Genomics Analysis and Visualization Platform, this is also the case for retinoblastoma tumor samples (Fig. 1). This database does not contain a similar analysis for SCLCs. However, it does contain expression data for other tumor types where the expression of CDA mRNA is not consistently low (e.g., melanoma, non-small cell lung carcinoma, and acute myeloid leukemia) (Fig. 1) for comparison with the expression of *CDA* in retinoblastoma samples. CDA converts cytidine into uridine. Therefore, a low level of *CDA* expression suggests a defect in the maintenance of the balance between cytosine nucleotides and the other pyrimidine nucleotides [26]. One major difference between retinoblastoma and SCLC is that whereas TP53 is mutated in most SCLCs [25], it is functional in most retinoblastomas although the activity of p53 may be weakened by MDM2 or MDM4 overexpression [27]. Moreover, as previously described [28] and as shown by our results below, p53 mutation in retinoblastoma may be associated with metastasis.

Preclinical studies on pharmacologic DHODH inhibition have shown efficacy against neuroblastoma and medulloblastoma, which are also pediatric cancers [7,28,29]. *MYCN* overexpression is also frequent in neuroblastoma, p53 is normally wild-type and these cells are deficient for *CDA* expression [26]. Medulloblastoma cell lines (depmap portal) and tumors (Fig. 1) also show low levels of CDA mRNA expression.

Furthermore, aside from presenting low levels of *RB1* expression, retinoblastoma cells show low levels of the mRNAs for the *RB1* paralog *RBL2* (Fig. 1), suggesting that the G1/S restriction point is severely impaired. This is strengthened by the observation that the mRNAs for E2F1 and E2F2, cyclins A1, A2 and cyclins E1 and E2 as well as the mRNAs for the ribonucleotide synthase subunits RRM1 and RRM2 are high in retinoblastoma primary tumors (Supplementary Fig. 1). Finally, another feature of retinoblastoma is that TYMS mRNA levels are high, suggesting that these tumor cells rely on dUMP, and therefore on UMP for the synthesis of dTTP (Supplementary Fig. 1).

### 3.2 Targeting DHODH in the *de novo* pyrimidine ribonucleotide synthesis pathway has a limited effect on retinoblastoma cells

**Fig. 2:**
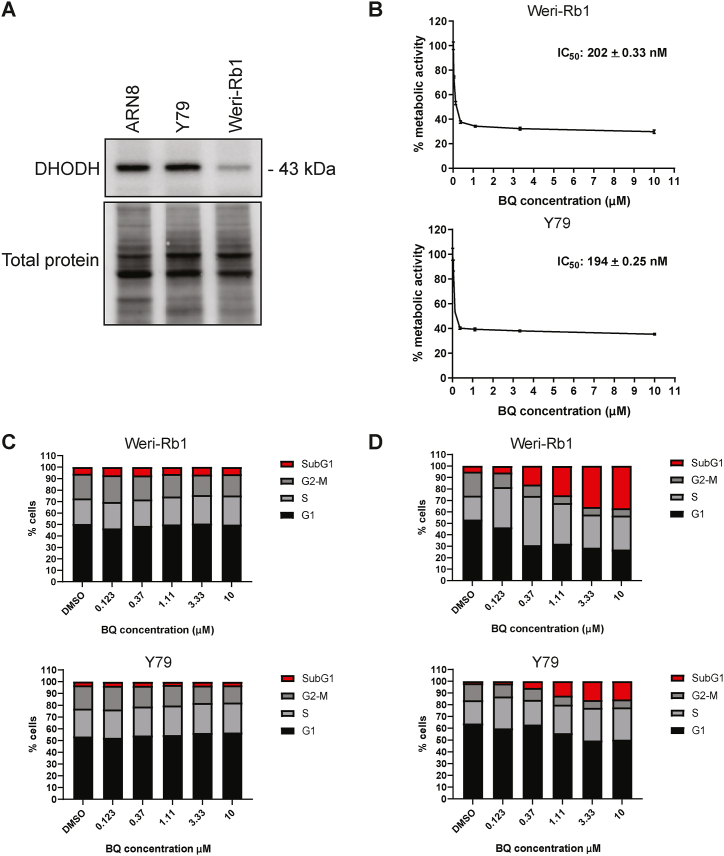
DHODH is expressed in retinoblastoma cells and its inhibition by BQ weakly affects cell growth. **A)** The protein expression of DHODH was analyzed by western blot in the Weri-Rb1 and Y79 cell line using ARN8 melanoma cell line as a positive control. Total protein was used as loading control. See Supplementary Material for uncropped images. **B)** WST-1 assay was performed on Weri-Rb1 and Y79 cell cultures after 3 days of treatment with BQ at the concentrations indicated. Graphs are representative of 4 biological repeats. IC50 values are given as weighted mean + SD. **C-D** Propidium iodide flow cytometry assays were performed after treating the Weri-Rb1 cells or Y79 cells with BQ for 3 days (**C**) and 6 days (**D**) at the indicated concentrations.

To assess whether DHODH would be a target to consider in retinoblastoma, first we selected two of the most commonly studied retinoblastoma cell lines (Weri-Rb1 and Y79). Fig. 2A shows that both cell lines express DHODH and that the expression of the enzyme is lower in the Weri-Rb1 cells. Next, we used the WST-1 assay to assess cell metabolic activity upon inhibition of DHODH with brequinar (BQ), a well-established DHODHi [30]. Upon treatment with increasing concentrations of BQ for 3 days, we observed a partial decrease in cell metabolic activity for both cell lines (Fig. 2B) with no changes in cell cycle progression (Fig. 2C, Supplementary Fig. 2A). Considering the slow growth of Weri-Rb1 and Y79 retinoblastoma cells in culture [31,32], we decided to increase the treatment time to 6 days using the same concentrations of BQ. Both cell lines responded to treatment at this time point. Gradual increases in the proportion of S phase cells as well as in the proportion of cells with a <2 N DNA content (SubG1 cells) were observed (Fig. 2D and Supplementary Fig. 2B). In this assay, Weri-Rb1 cells were more sensitive to treatment than Y79 cells perhaps due to the higher levels of DHODH in Y79 cells and/or the fact that Weri-Rb1 cells are grown in medium with 10 % FBS whereas Y79 cells were grown in medium with 20 % FBS. The uridine content in FBS is in the 5 μM range [33]. However, even in the Weri-Rb1 cultures, only about 40 % of the cells died, suggesting that DHODHi as single agents are not likely to be efficacious against retinoblastoma.

### 3.3 The uridine uptake inhibitor dipyridamole does not affect retinoblastoma cell growth

**Fig. 3:**
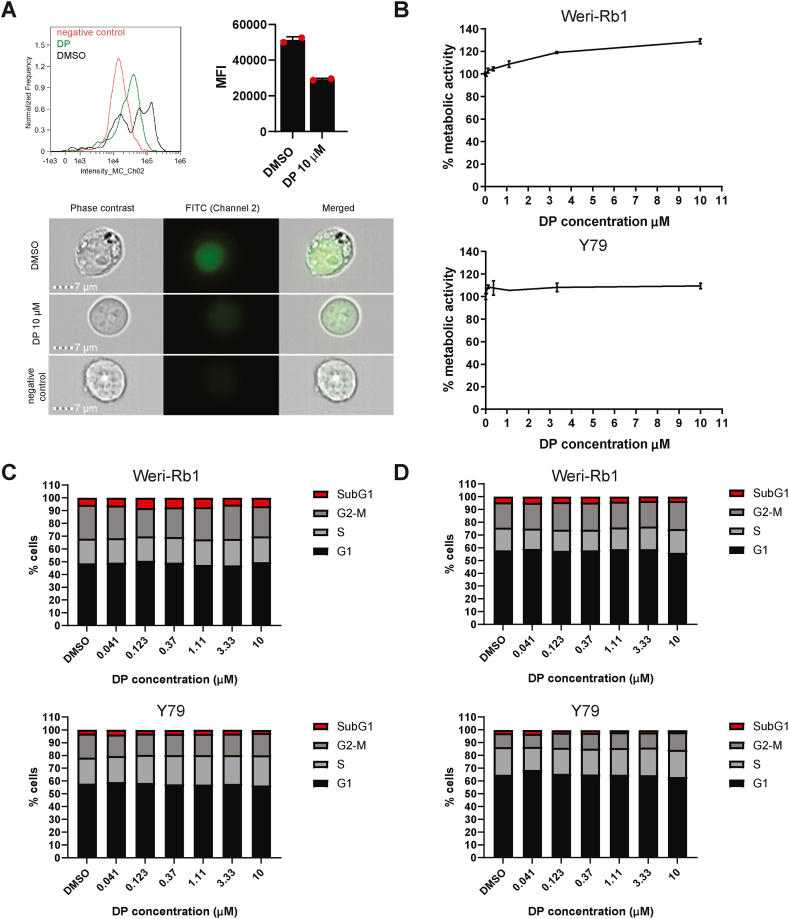
DP inhibits uridine uptake in retinoblastoma cells and does not affect retinoblastoma cell growth. **A)** Weri-Rb1 cells were treated with DMSO or 10 μM DP in serum replacement medium. After 24 h, 5-ethynyl uridine was added for 1 h. Cultures without 5-ethynyl uridine pulse served as negative controls. Cells were fixed and processed for the click chemistry reaction before performing imaging flow cytometry. Normalized frequency (upper left) and mean fluorescence intensity values (upper right) are plotted for two technical replicates. Representative images are shown. **B)** WST-1 assay was performed for the Weri-Rb1 and Y79 cell lines after 3 days of treatment with DP at the concentrations indicated. Graphs are representative of 4 biological repeats. **C-D** Propidium iodide flow cytometry was performed by treating the Weri-Rb1 cells and Y79 cells treated with the indicated concentrations of DP for 3 days (**C**) and 6 days (**D**). The proportion of cells at different phases of the cell cycle are shown.

Next, we tested whether blocking of the salvage pathway of pyrimidine ribonucleotide synthesis would affect retinoblastoma cells. To investigate this, we used the nucleoside transport inhibitor dipyridamole (DP), which is known to block uridine uptake from the extracellular environment through inhibition of human nucleoside equilibrative transporter 1 (hENT1) and to a lesser extent, by inhibition of hENT2 [34,35]. First, we verified the uridine uptake inhibitory activity of DP by treating the ARN8 melanoma cell line. In agreement with previous studies, DP was able to decrease cellular ^3^H-uridine uptake at low nanomolar concentrations (Supplementary Fig. 3A). Additionally, to confirm whether DP could block uridine uptake in retinoblastoma cells, we treated the Weri-Rb1 cells with DMSO or 10 μM DP for 24 h followed by a 1 h pulse of 5-ethynyl uridine. After performing a click chemistry reaction with Alexa Flour 488 azide, we detected uridine uptake activity using imaging flow cytometry. Both normalized frequency and mean fluorescence intensity data of 5-ethynyl uridine showed a reduction of uridine uptake by DP treated cells relative to DMSO (Fig. 3A).

To check whether DP as a single agent could affect the growth of retinoblastoma cells, we treated Weri-Rb1 and Y79 cells with DMSO or increasing concentrations of DP for 3 days and performed WST-1 assays. DP did not alter cell metabolic activity in either cell line (Fig. 3B). Furthermore, no changes were observed on cell cycle distribution after 3 days or 6 days of treatment with DP (Fig. 3C and D and Supplementary Figs. 3B and C).

### 3.4 Dipyridamole synergizes with brequinar to promote retinoblastoma cell death

The logical follow-up study was to investigate whether simultaneously blocking both the *de novo* and the salvage pathways of pyrimidine ribonucleotide synthesis would promote cell death. We used two different concentrations of BQ, 200 nM and 1 μM based on the IC_50_ values and lowest concentration of BQ that led to maximum reduction of metabolic activity in the Weri-Rb1 and Y79 cell lines (Fig. 2B). Since DP did not have any effect on metabolic activity (Fig. 3B), we decided to combine BQ with wide range of DP concentrations and analyze the cell cycle profiles after 3 days (Supplementary Figs. 4A and B). With both concentrations of BQ used, there was an increase in S phase accumulation of cells and an increase in the proportion of cells with a SubG1 DNA content with increasing concentration of DP. From these, results we selected 1 μM DP for subsequent experiments.

**Fig. 4:**
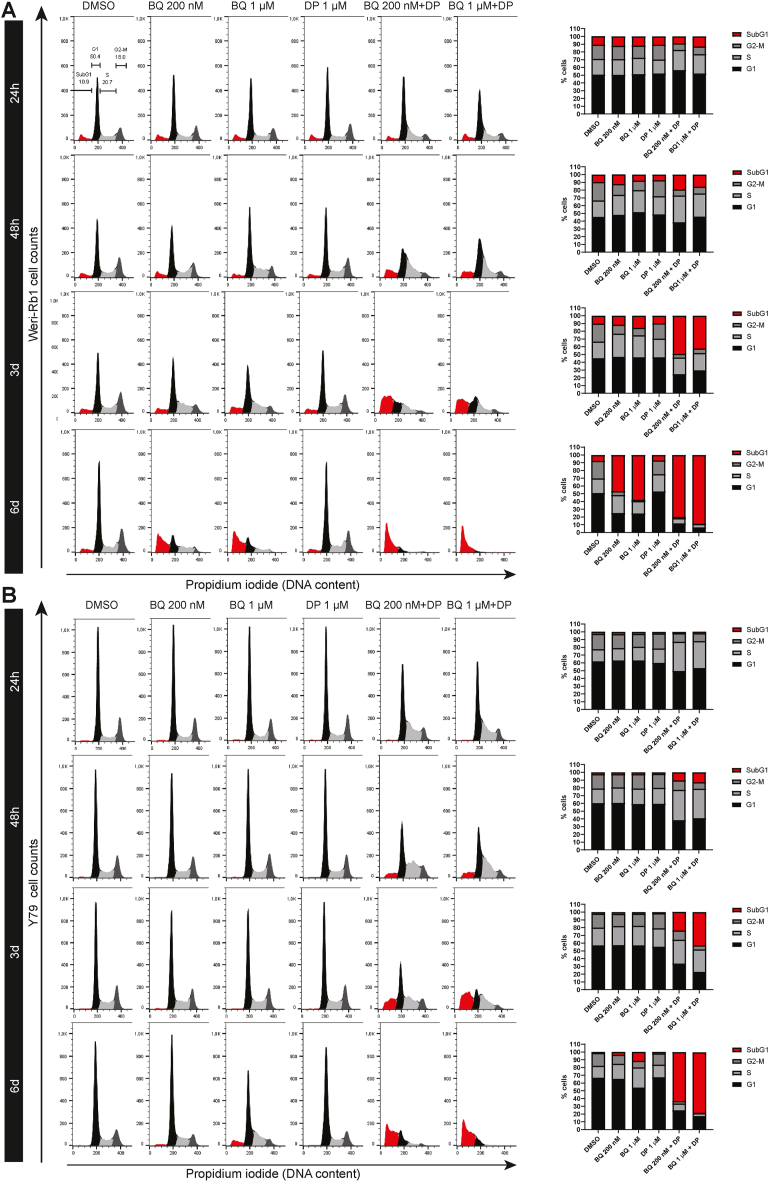
DHODH inhibition in combination with uridine uptake blockage causes S phase accumulation followed by cell death. Propidium iodide flow cytometry analysis of Weri-Rb1 (**A**) and Y79 (**B**) cells treated as indicated for 24 h, 48 h, 3 days and 6 days. Bar graphs representing the different phases of the cell cycle are on the right.

Using the above-mentioned compound concentrations, we studied retinoblastoma cell cycle progression over time. In the Weri-Rb1 cell line (Fig. 4A), combination treatments caused S phase accumulation at 24 h. This was more pronounced at 48 h, when cells also started to die. By 3 days, the combinations markedly increased the proportion of SubG1 cells and at 6 days, up to 90 % of the cells were dead. A similar sequence of events was also observed for the Y79 cell line (Fig. 4B).

**Fig. 5:**
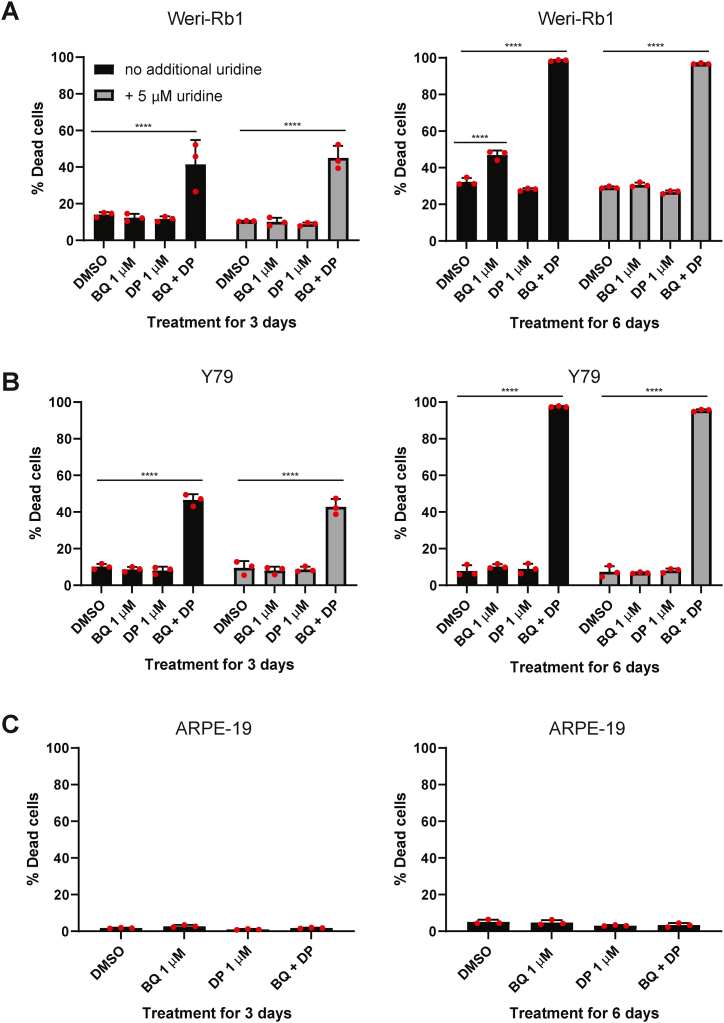
The combination treatment is not impaired by supplementation with 5 μM uridine. Weri-Rb1 (**A**) and Y79 (**B**) and cells were treated as indicated in presence or absence of 5 μM uridine for 3 days or 6 days. A live/dead marker was used to analyze the % of dead cells using flow cytometry. **C)** ARPE-19 cells were treated as indicated for 3 days or 6 days and analyzed as above. All data shown are given as mean ± SD for three technical replicates and are representative of 3 biological repeats. One-way ANOVA followed by Dunnette's multiple comparison test was performed to compare between the groups, ****p < 0.0001.

Since the presence of uridine can weaken the effect of DHODHi [11], we tested whether the addition of uridine reduce the cell death promoting effects of the combination treatment. As it was we could not find published data on uridine levels in the human vitreous humor, a concentration of 5 μM uridine was selected based on human serum uridine levels [33]. Weri-Rb1 and Y79 cells were treated with DMSO, BQ 1 μM, DP 1 μM and BQ + DP in absence or presence of 5 μM uridine for 3 days and 6 days. Cell death was analyzed by flow cytometry using a live/dead marker. In case of the Weri-Rb1 cells (Fig. 5A), a 3-day treatment with BQ + DP resulted in 41.5 ± 13.3 % cell death. In the presence of 5 μM uridine, cell death with the combination treatment was similar (45 ± 6.6 % cell death). On day 6, cell death upon combination treatment increased to 98.5 ± 0.4 %, whereas treatment with 1 μM BQ on its own caused 46.9 ± 2.5 % cell death. Both of these treatments led to a significantly higher level of cell death (p < 0.0001) compared to DMSO controls (15.5 ± 2.2 %). In the presence of uridine, the cell death effect of the combination was unchanged at 96.7 ± 0.3 % compared to 29.2 ± 0.6 % for DMSO + uridine (p < 0.0001). As expected, the presence of uridine reduced the activity of BQ as a single agent. A similar trend for the combination treatment was observed also for the Y79 cell line (Fig. 5B).

To further confirm that DP is inhibiting uridine uptake in these assays, Weri-Rb1 cells were treated with using serum free medium (serum replacement 3 medium) in the absence or presence of 5 μM uridine for 3 days and cell death was measured. The rationale for using serum free medium was that the FBS used in standard cell culture conditions contains uridine [33]. As shown in Supplementary Fig. 5, in the absence of uridine, both BQ and the combination caused similar increases in cell death. Upon supplementation with 5 μM uridine, the combination treatment continued to cause cell death whereas BQ as a single agent lost its effect. Taken together, these data indicated that DP indeed inhibits uridine uptake.

In order to evaluate that the treatments do not cause extensive cell death in normal cells, the ARPE-19 retinal pigment epithelium cell line was treated compounds alone or in combination for 3 and 6 days. None of the treatment conditions showed extensive cell death relative to DMSO control levels (Fig. 5C).

### 3.5 Using another DHODHi and another uridine uptake inhibitor confirms the synergistic effect of blocking both pyrimidine ribonucleotide synthesis pathways

To confirm that retinoblastoma cell death is actually due to inhibition of the *de novo* and salvage pyrimidine ribonucleotide pathways, we tested BAY2402234, a novel DHODHi [36] and the established nucleoside transport inhibitor NBMPR [34]. In Supplementary Fig. 6A, Weri-Rb1 cells were treated for 3 days with BAY2402234 and DP as single agents and in combination. These cells were also treated with BQ and NBMPR as single agents and in combination for 3 days (Supplementary Fig. 6B). As described for DP on its own, NBMPR alone did not have any effect on cell cycle profiles. The 3-day treatments with either BAY2402234 or BQ on their own did not have an effect either. Instead, there was an increase the proportion of cells with a SubG1 DNA content with the combination treatments.

### 3.6 DHODH inhibition in combination with uridine uptake blockage activates apoptosis in retinoblastoma cells

**Fig. 6:**
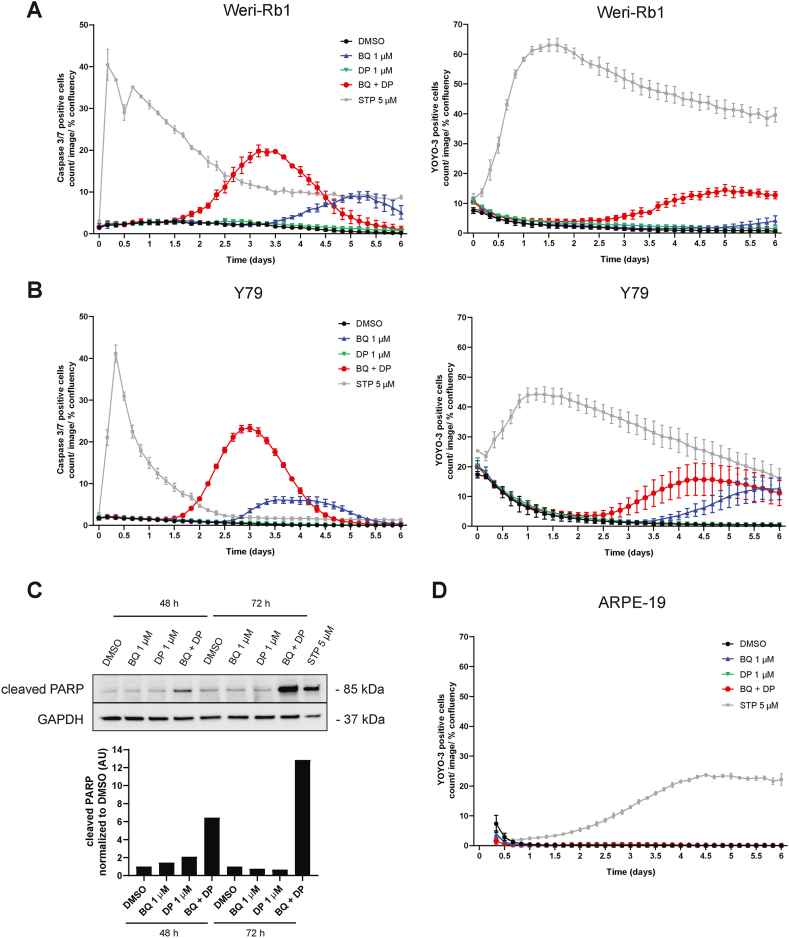
Retinoblastoma cell death associates with caspase 3/7 activation. Live cell imaging of Weri-Rb1 cells (**A**) or Y79 cells (**B**) treated as indicated for 6 days in presence of a substrate for caspase 3/7 (left) or the cell impermeable nuclear dye YOYO-3 (right) as described in Methods. Data corresponds to the mean + SD for 3 technical replicates and is representative of 3 biological repeats. Stauropsorine (STP) is used as positive control. **C)** Cleaved PARP-1 was analyzed by western blot after treating the Weri-Rb1 cells as indicated. Staurosporine (STP) was used as positive control and GAPDH as a loading control. Bar graph shows cleaved PARP-1 expression normalized to the DMSO treated sample. See Supplementary Material for uncropped images. **D)** Live cell imaging was performed for ARPE-19 cells treated as indicated for 6 days in the presence of YOYO-3 as described in Methods. Data corresponds to the mean + SD for 3 technical replicates and is representative of 3 biological repeats.

It has been previously shown that DHODH inhibition leads to apoptosis mediated cell death in cancer cells [11,12,37]. To test whether the same held true for retinoblastoma cell lines, we treated Weri-Rb1 and Y79 cells with compounds alone or in compound combination. In addition, we included either a caspase-3/7 substrate, which upon cleavage by activated caspases 3 and 7 emits a fluorescent signal, or the impermeable nuclear dye YOYO-3 which monitors cell death upon loss of cell membrane integrity. Staurosporine was used as a positive control. In the case of the Weri-Rb1 cell line (Fig. 6A), caspase 3/7 signal increased after 1.5 days and peaked at around 3 days of treatment with the compound combination. The YOYO-3 signal started to rise after the caspase signal at around 3 days. BQ as a single agent also activated caspase 3/7 at around 3.5 days of treatment, albeit the signal was not as strong as with the combination. The YOYO-3 signal for BQ single treatment rose slightly after 5 days. Real time imaging videos taken with the phase contrast feature of the IncuCyte S3 system showed that the Weri-Rb1 cells treated with the combination formed chain clusters in the early stages of the treatment, but unlike the DMSO and DP treated cells, only small clusters were formed. After 3 days of treatment, both BQ and combination treated cells lost their ability to form these chains and became rounder and more condensed (Supplementary Fig. 7 and Supplementary video files), which was indicative of cell death. For DMSO and DP treated cells, larger clusters and chains were formed over time (Supplementary Fig. 7A and Supplementary video files). A similar pattern was seen for the Y79 cell line (Fig. 6B).

Taken together, these results suggest that retinoblastoma cell death by pyrimidine ribonucleotide synthesis inhibition is due to caspase mediated apoptosis. Accordingly, western blot analysis (Fig. 6C) revealed an increase in cleaved poly (ADP-ribose) polymerase-1 (PARP-1) protein, which is characteristic of apoptosis and due to the activity of caspases 3 and 7 [38].

To ensure that the treatments did not cause extensive cell death in normal cells, we performed also live cell imaging for the ARPE-19 cell line using the same compound treatments as above and the YOYO-3 nuclear dye. Other than staurosporine, none of the treatments led to any noticeable increase in the YOYO-3 signal (Fig. 6D). However, ARPE-19 cells treated with the combination (see Supplementary video files) proliferated less compared to DMSO, BQ and DP, all of which became confluent by the end of the 6-day timepoint (Supplementary Fig. 7C). Here it may be relevant to mention that ARPE-19 cells, unlike the Weri-Rb1 and Y79 cells, grow as adherent cultures, and we cannot exclude that this difference influences the onset of apoptosis.

### 3.7 DHODH and uridine uptake inhibition can promote retinoblastoma cell death irrespective of p53 status

Retinoblastoma cell lines are difficult to grow in culture and even the most frequently used ones (Weri-Rb1 and Y79) grow slowly. In agreement with our experience, according to the Expasy website (https://www.cellosaurus.org/) the doubling time for Weri-Rb1 cells is between 49 and 96 h and for Y79 cells it is between 40 and 52 h. This is of importance with regards to inhibitors of nucleotide synthesis as these are bound to primarily affect rapidly proliferating cells. Nevertheless, Weri-Rb1 and Y79 cells were killed effectively by simultaneously inhibiting the *de novo* and the salvage pyrimidine nucleotide synthesis pathways.

**Fig. 7:**
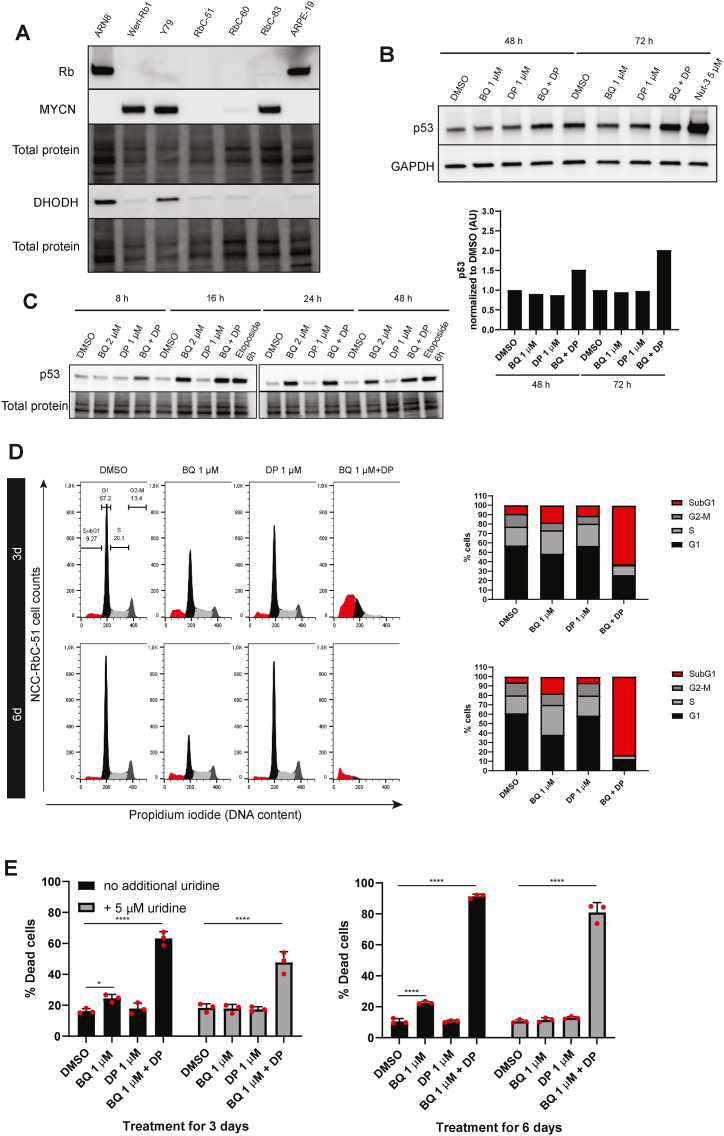
The effect of DHODHi in combination with dipyridamole induces p53 in wild-type tumors but its killing effect is not dependent on p53. **A)** Western blots for different retinoblastoma cell lines. ARN8 melanoma and ARPE-19 cells were used as controls. **B–C** p53 protein levels were analyzed by western blot after treating the Weri-Rb1 cells or Y79 cells as indicated. Nutlin-3 (Nut-3) or etoposide were used as positive controls. See Supplementary Material for uncropped images. **D)** Propidium iodide flow cytometry was performed after treating NCC-RbC-51 cells as indicated for 3 days and 6 days. Cell cycle distribution is shown on the left, and the % of cells in each cell cycle phase is represented by a bar graph on the right. **E)** NCC-RbC-51 cells were treated as indicated in presence or absence of 5 μM uridine for 3 days or 6 days. A live/dead marker was used to analyze the % of dead cells using flow cytometry. Data is given as mean ± SD for three technical replicates and is representative of 3 biological repeats. One-way ANOVA followed by Dunnette's multiple comparison test was performed to compare between the groups; *p < 0.05, ****p < 0.0001.

To expand our studies, we purchased other retinoblastoma cell lines from RIKEN (NCC–RbC-39, NCC-RbC-51, NCC-RbC-54, NCC-RbC-59, NCC-RbC-60, NCC-RbC-67, NCC-RbC-83, NCC-RbC-92 and NCC-RbC-T1) and succeeded to grow three of them (NCC–RbC-51, NCC-RbC-60 and NCC-RbC-83) at sufficient levels for further experiments using medium supplementd with 20 % FBS. The doubling times for NCC-RbC-51 reported by Expasy is between 67 and 85 h and for NCC-RbC-60 cells it is around 47 h. The doubling time for NCC-RbC-83 cells is not reported by Expasy. However, and also in agreement with our experience, RIKEN recommends to subculture NCC-RbC-60 and NCC-RbC-83 cells by diluting 1:2–4 and only once per week. Western blots for these cell lines are in Fig. 7A.

Retinoblastoma tumors usually retain wild-type p53 and DHODHi have been shown previously to activate p53 in p53 wild-type cells [11]. Accordingly, for Weri-Rb1 and Y79 cells, increases in p53 expression were detected with the BQ + DP combination treatments (Fig. 7B and C). In these experiments, nutlin-3 (an MDM2 inhibitor that prevents p53 degradation) or the DNA damaging agent etoposide were used as positive controls, confirming that these cells express wild-type p53, as previously reported [39]. BQ on its own was able to increase p53 levels in Y79 cells, and the BQ + DP combination increased p53 levels as early as 8 h after treatment (Fig. 7C).

Nevertheless, it has been demonstrated that DHODHi can cause cell death also in p53 deficient cells [36]. Interestingly, when we sequenced *TP53* in the NCC-RbC-51 cell line, we observed that it was mutated (c.404G > T, p. Cys135Phe). Published cell based assays [40] confirm loss of function for this p53 variant. Our analysis showed also that the wild-type allele is lost, as only T was detected in position 404. NCC-RbC-51 cells were derived from a cervical lymph node metastatic site of a 4-year-old male with bilateral retinoblastoma. As shown in Fig. 7D, NCC-RbC-51 cells were treated with DMSO, BQ 1 μM, DP 1 μM or the compound combination. Cell cycle distribution analysis showed that BQ as a single agent was able to induce accumulation of cells in S phase as well as to increase in the percentage of cells with a SubG1 DNA content at both 3 days and 6 days of treatment. The compound combination caused a further increase in SubG1 levels at 3 days treatment, and by 6 days treatment most cells were dead.

Additionally, we checked whether the presence of physiologic levels of uridine could prevent the cell killing effect of the treatments. NCC-RbC-51 cells were treated as above in presence or absence of 5 μM uridine and the percentage of dead cells with a live/dead marker was determined (Fig. 7E). At the 6-day timepoint, BQ showed a similar response as the 3-day timepoint, where cell death was higher (22.5 ± 0.84 %) relative to DMSO control (10.5 ± 1.8), and the effect was lost in presence of uridine. The compound combination caused cell death to a much higher extent (91.4 ± 1.3 %) relative to control and was still high (81 ± 6.3 %) in presence of uridine.

As previously determined, NCC-RbC-60 and NCC-RbC-83 cells express functional p53 [41]. Unlike Weri-Rb1, Y79 and NCC-RbC-83 cells, the NCC-RbC-60 and NCC-RbC-51 cell lines showed low levels of MYCN protein (Fig. 7C), and NCC-RbC-83 expressed low but detectable levels of Rb. The killing effect of the BQ + DP on NCC-RbC-60 and NCC-RbC-83 at 3 and 6 days was weaker than in the other cell lines (Supplementary Fig. 8), although a marked accumulation of cells in S phase was evident in NCC-RbC-83 at 3 days followed by an increase in cell death at 9 days. Noteworthy, treatment with BQ alone as well as BQ + DP reduced MYCN protein levels over time in Y79 cells (Supplementary Fig. 9). Further analyses of more cell lines grown using identical culture medium and %FBS will be required to test whether p53 status and MYCN protein levels influence outcome.

### 3.8 Pyrimidine ribonucleotide synthesis genomic alterations in a 102-retinoblastoma cohort

Analyzing recently published data on genomic alterations related to annotated genes for a 102-cohort [23], reveals that loss of chr16q22.2 (containing *DHODH*) is amongst the most frequent alterations in retinoblastoma and comparable to *MYCN* and *MDM4* gain/amplification (Supplementary Table 1). Following the proposed classification of the 102 retinoblastomas [23], 4 of 38 subtype 1 retinoblastomas, 33 of 58 subtype 2 retinoblastomas and 5 of 6 subtype 3 retinoblastomas show chr16q22.2 loss. Chr16q22.2 gains were detected in 3 subtype 1 retinoblastomas. According to previous data, clones selected for their resistance to brequinar, overexpressed DHODH [42]. These two observations suggest that a wide range of retinoblastomas could be sensitive to low concentrations of DHODH inhibitors. Of note, subtype 2 retinoblastomas have a higher propensity to metastasis than subtype 1.

Also of interest, according to the genomic alterations in the 102-retinoblastoma cohort [23], retinoblastomas often show gains in genes expressing factors involved in the pyrimidine ribonucleotide salvage pathway and in particular, gains in chr6p21.1 (containing *SLC29A1* gene for the ENT1 uridine transporter) and/or gains in genes expressing uridine kinases (chr1q24.1, including *UCK2* and chr20q13.33, including *UCKL1*). Uridine kinases are necessary for the synthesis of UMP [10]. 19 of 38 subtype 1 retinoblastomas, 51 of 58 subtype 2 retinoblastomas and 5 of 6 subtype 3 retinoblastomas show gains in genes encoding ENT1 and/or uridine kinases (Supplementary Table 1). Furthermore, *SLC29A1* is significantly less methylated in subtype 2 than in subtype 1 retinoblastomas [23]. This suggests that inhibition of the salvage pathway may be more frequently necessary to kill subtype 2 retinoblastomas than to kill subtype 1 cells. Interestingly, 2 of the 102 retinoblastomas showed a homozygous deletion for *DHODH*, suggesting that these two cancers may respond to inhibitors of uridine transport on their own.

**Fig. 8:**
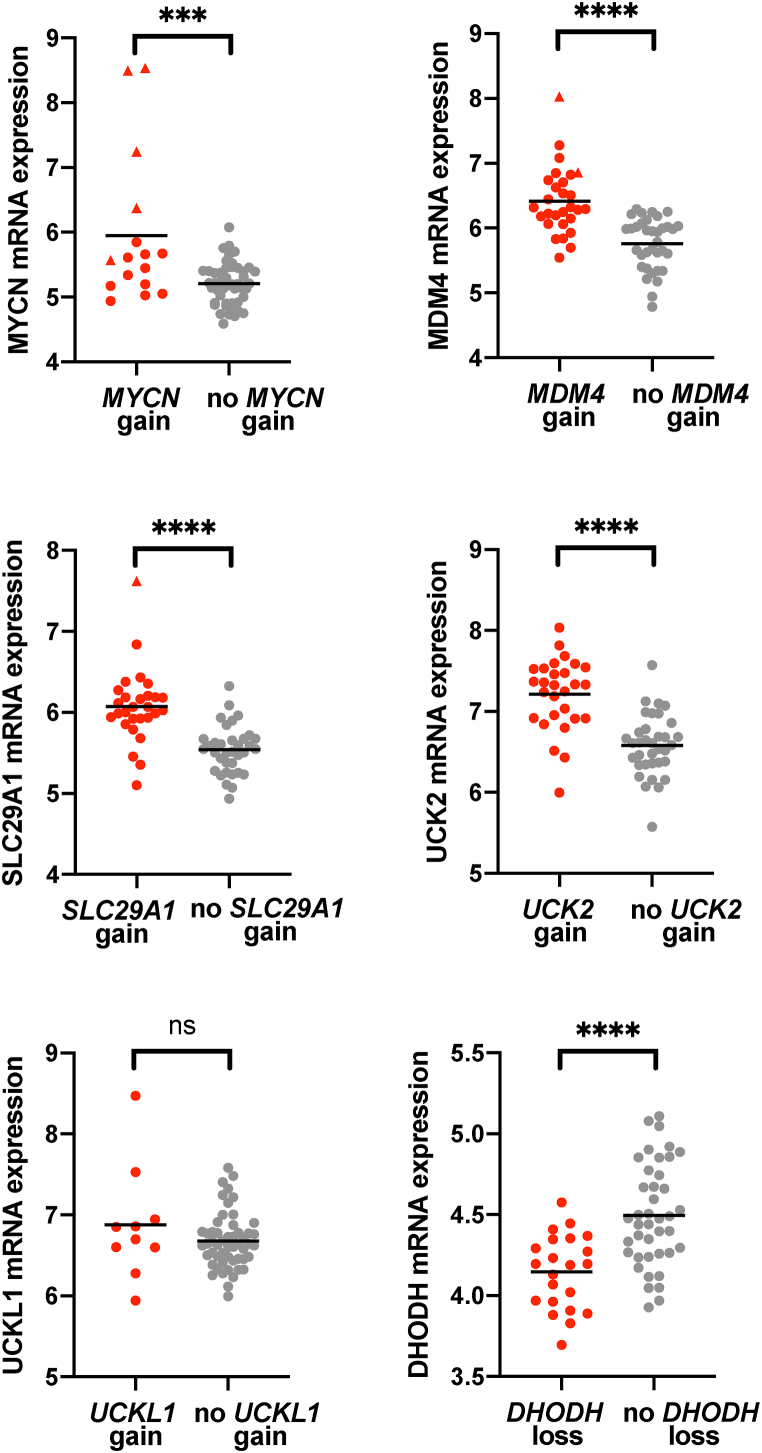
Analysis of the correlation between gain/loss and expression of the indicated genes according to the transcriptomic data for retinoblastomas available in GSE58780 [23]. Triangles indicate samples where the relevant gene is amplified. Lines indicate mean values and p values were obtained by unpaired t tests (***p < 0.001, ****p < 0.0001).

Finally, using the transcriptomic data available in GSE58780 [23], we determined whether the expression of *MYCN*, *MDM4*, *SLC29A1*, *UCK2, UCKL1* and *DHODH* genes correlates with their loss or gain. Fig. 8 shows that DHODH expression is lower in the tumors where there is loss of *DHODH* gene and that *MYCN*, *MDM4*, *SLC29A1* and *UCK2* expression is higher in the samples that show gains for *SLC29A1* and *UCK2*. Instead, *UCKL1* expression did not show correlation. This could be due to the fact that *UCKL1* loss also affects its antisense gene *UCKL1-AS* (Supplementary Table 1).

## 4 Discussion

DHODHi are effective in preclinical models for SCLC, neuroblastoma and medulloblastoma, and as described here, these cancers share some molecular features with retinoblastoma (Fig. 1). Furthermore, the neuroblastoma study showed that DHODH blockage in combination with temozolomide (an alkylating agent) can lead to cure in a murine model. The work on medulloblastoma is also of special interest because, together with preclinical studies on glioma [8], it demonstrated that a DHODHi, BAY2402234, passes the blood brain barrier. Hence, it is possible that some DHODHi also pass the blood-retinal barrier if they are to be administered systemically. For all these reasons, we decided to evaluate whether targeting pyrimidine ribonucleotide synthesis would be an attractive strategy to consider for the local or systemic treatment of retinoblastoma patients.

One major challenge was that retinoblastoma cells grow very slowly. Indeed, as shown here, the effect of DHODHi as single agents is slow, and even weak. Therefore, reducing pyrimidine ribonucleotide synthesis more radically seemed necessary in the case of retinoblastoma. Combination of the DHODHi BQ with the nucleoside transport inhibitor DP enhanced the effect of BQ and caused a significant increase in cell death which at least in part is due to apoptosis. Importantly, the results obtained with BQ were equivalent to the results obtained with another DHODHi, BAY2402234. Additionally, the effect of DP could be mimicked by NBMPR, another inhibitor of nucleoside uptake. This supports that the effects of BQ or DP in the assays presented here are due to blockage of DHODH or nucleoside uptake, respectively. Nevertheless, it is relevant to discuss here the current knowledge on the specificity of the compounds used in this study.

Strong evidence supporting the specificity of BQ and BAY2402234 for DHODH comes from the fact that the effect of BQ and BAY2402234 on cell proliferation/viability are completely prevented by the addition of an excess of uridine and the observation that cells become resistant to brequinar by acquiring increase the expression of DHODH [20,36,42]. Accordingly, and as shown here for retinoblastoma, the effect of BQ on retinoblastoma cells is weakened by the addition of uridine to the medium.

The ability of DP to target nucleoside uptake is well documented uptake [19,20,34,35] and confirmed in this work. We also show that DP on its own does not have any effect with regards to the proliferation/viability of retinoblastoma cells even a 10 μM, which is 10-fold the concentration used in the experiments combining DP with DHODH inhibitors. DP's effect only became detectable when used in combination with a DHODHi. Furthermore, DP ablated the ability of 5 μM uridine to rescue retinoblastoma cells from DHODH inhibition but had no significant effect when uridine was completely absent. In addition, and as shown by others [19,20], very high concentrations of uridine (50–100 μM), can markedly diminish the effect of the DP + DHODHi combination on the viability of cells. This is expected because at very high concentrations, uridine may prevent DP binding to pyrimidine nucleoside transporters or become internalized by DP-insensitive transporters. Taken together, these data indicated that DP inhibits uridine uptake by retinoblastoma cells.

However, despite this data, we cannot exclude that DP influences other events aside from nucleoside transport and whether these affect the proliferation/viability experiments performed here. Indeed, DP is thought to inhibit phosphodiesterases that degrade nucleoside 3′,5′-cyclic monophosphates (cNMPs) to NMPs [43]. Hence, DP could be influencing NMP levels through an additional mechanism. However, as stated above, another validated nucleoside transport inhibitor (NBMPR) had the same effect on our assays as DP, suggesting that nucleoside import inhibition by DP does play a role in studies using the DP in combination with DHODHi [19,20]. The reason we chose to use DP for most of the studies is that unlike NBMPR, DP has been tested for ocular disorders (intravitreally and topically). We believe that this is important from a from a clinical perspective. However, from a mechanistic perspective, it will be necessary to test whether knockdown and overexpression of nucleoside transporters and/or of phosphodiesterases influences the response to pharmacologic inhibition of DHODH alone and in combination with DP. Interestingly, cUMP is degraded to UMP by one of the DP-sensitive phosphodiesterases (PDE3A) [44]. This suggests that DP could reduce UMP levels (the central molecule in pyrimidine nucleotide metabolism [10]), and therefore the levels of all pyrimidine nucleotides, by blocking uridine uptake as well as by blocking phosphodiesterase activity.

Chemotherapeutic agents and radiation used today for the treatment of retinoblastoma have been reported to cause damage to the unaffected regions of the retina [6]. In the experiments reported here, ARPE-19 cells, which are derived from retinal pigment epithelium cells, did not die with any of the treatments. However, live cell imaging analysis, revealed that ARPE-19 cells treated with the combination proliferated less. According to some studies, the retinal pigment epithelium undergoes a sharp diminishment in cell proliferation soon after birth, as do most cell types present in the neuroretina [[45], [46], [47]]. This may mean that the retinal pigment epithelium will not be affected by the compound treatments proposed here. Clinically approved DHODHi teriflunomide, for treatment of multiple sclerosis, and its prodrug leflunomide, for the treatment of rheumatoid arthritis have not been reported to cause blindness or visual impairment and there have been no such reports with brequinar or BAY2402234 to the best of our knowledge. Moreover, a recent report on the intravitreal injection of another novel DHODHi (PP-001/KIO-100) for the treatment of uveitis, did not have any effect on retinal pigment epithelium cells [48] and has proven ocular safety in phase I clinical trial [49]. Dipyridamole has also been previously investigated as a treatment for ocular disorders and has been administered both intravitreally and topically [50]. Furthermore, DP is used systemically to prevent blood clots. Taken together, this indicates that the treatment strategy used in this study should not pose severe visual impairment.

Despite the difficulties in growing retinoblastoma cells, we were able to culture several retinoblastoma cell lines that demonstrated different DHODH and MYCN protein levels, and *TP53* status. Here, we show that NCC-RbC-51 cells, which derive from a retinoblastoma tumor in a metastatic site, express mutant p53 and that they responded to BQ as a single agent as well as in combination with DP. Thus, this treatment strategy may not only hold merit for p53 wild-type retinoblastomas, but also difficult to treat metastasized tumors that may express mutated p53. *MYCN* overexpression is associated with increased malignancy in numerous tumor types and amplification of this oncogene is one of the most often events in retinoblastoma [51]. However, due to the difficulties in growing retinoblastoma cell lines, a limitation of this study is that further assessments are necessary to conclude whether MYCN protein levels influence the killing effect of complete blockage of the pyrimidine ribonucleotide synthesis pathways.

Underlining the vulnerability of retinoblastoma cells to pyrimidine nucleotide synthesis inhibitors, we have shown that loss of chr16q22.2 (containing the *DHODH* gene) and gains in chromosome regions expressing pyrimidine ribonucleotide salvage factors (uridine transporter ENT1 and two uridine kinases) are frequent in metastatic retinoblastoma. Furthermore, mRNA levels of *DHODH*, *SLC29A1* (ENT1) and *UCK2* correlate with chromosome region gains or losses.

In summary, this study illustrates that targeting the *de novo* and salvage pyrimidine ribonucleotide synthesis pathways simultaneously may be an effective approach to promote retinoblastoma cell death. However, this study is limited to cell culture conditions. Further investigations *in vivo* or with organotypic models need to be performed to assess treatment efficacy and possible side effects of local and systemic treatments. Finally, we point out that blockage of pyrimidine ribonucleotide synthesis should not be seen only as an alternative to melphalan. By causing the accumulation of retinoblastoma cells in S phase, pyrimidine ribonucleotide synthesis blockage may enhance the effects of melphalan selectively, that is without increasing the toxicity of melphalan on normal non-proliferating tissues of the eye. This may allow the use of lower doses of melphalan and impact in reducing the need of eye enucleation in children afflicted with retinoblastoma.

## Data availability statement

Data included in article/supp. material/referenced in article. Further raw data can be made available upon request. The article does not include new databases relevant for a public repository.

## Ethics statement

This work only includes previously published data derived from human specimens.

This work does not include experiments with animals.

## Funding statement

This research was funded by grants from the Swedish Cancer Society (22 2493Pj, 19 0096Pj), the Swedish Research Council (2017-02041, 538-2013-8807), the Swedish Childhood Cancer Fund (2019-0089, 2022-0093), the Radiumhemmet Funds (191172), and Karolinska Institutet. Open access funding is provided by Karolinska Institutet.

## CRediT authorship contribution statement

**Tanzina Mollick:** Writing – original draft, Visualization, Validation, Methodology, Investigation, Formal analysis, Data curation, Conceptualization. **Suhas Darekar:** Writing – review & editing, Investigation, Conceptualization. **Basile Dalarun:** Writing – review & editing, Investigation. **Flavia Plastino:** Writing – review & editing, Investigation. **Juan Zhang:** Writing – review & editing, Investigation. **Andres Pastor Fernández:** Writing – review & editing, Investigation. **Twana Alkasalias:** Writing – review & editing, Investigation. **Helder André:** Writing – review & editing, Conceptualization. **Sonia Laín:** Writing – review & editing, Supervision, Resources, Project administration, Methodology, Investigation, Funding acquisition, Data curation, Conceptualization.

## Declaration of competing interest

The authors declare the following financial interests/personal relationships which may be considered as potential competing interests:S.L. is an inventor of patent no. WO/2017/077,280 on a DHODH inhibitor class not used in this study and owns stocks in Genase Therapeutics BV.

## Appendix A Supplementary data

Multimedia component 2 Multimedia component 2

Multimedia component 3 ARPE19 1uMBQ+1uMDP

Multimedia component 4 ARPE19 DMSO

Multimedia component 5 WeriRb11uMBQ+1uMDP

Multimedia component 6 WeriRb1 DMSO

Multimedia component 7 Y79 1uMBQ+1uMDP

Multimedia component 8 Y79 DMSO
