# Supplementary material for: Retinoblastoma vulnerability to combined de novo and salvage pyrimidine ribonucleotide synthesis pharmacologic blockage
Source: Heliyon. 2023 Dec 17;10(1):e23831. doi: 10.1016/j.heliyon.2023.e23831 (PMC10851301; doi:10.1016/j.heliyon.2023.e23831)

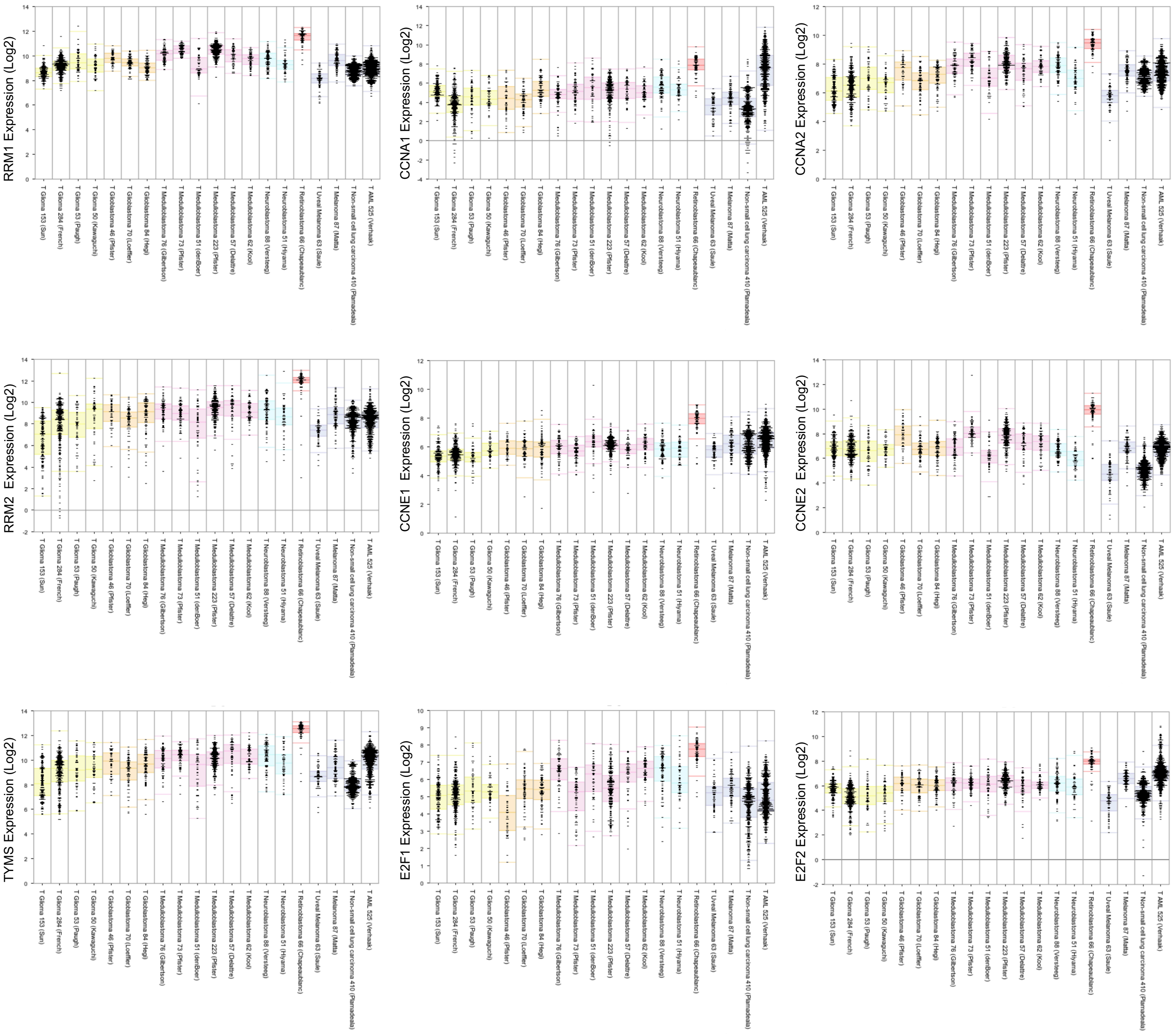

Supplementary Fig. 1. Expression of RRM1, RRM2, TYMS, CCNA1, CCNA2, CCNE1, CCNE2, E2F1 and E2F2 in tumors analyzed as in Fig. 1.

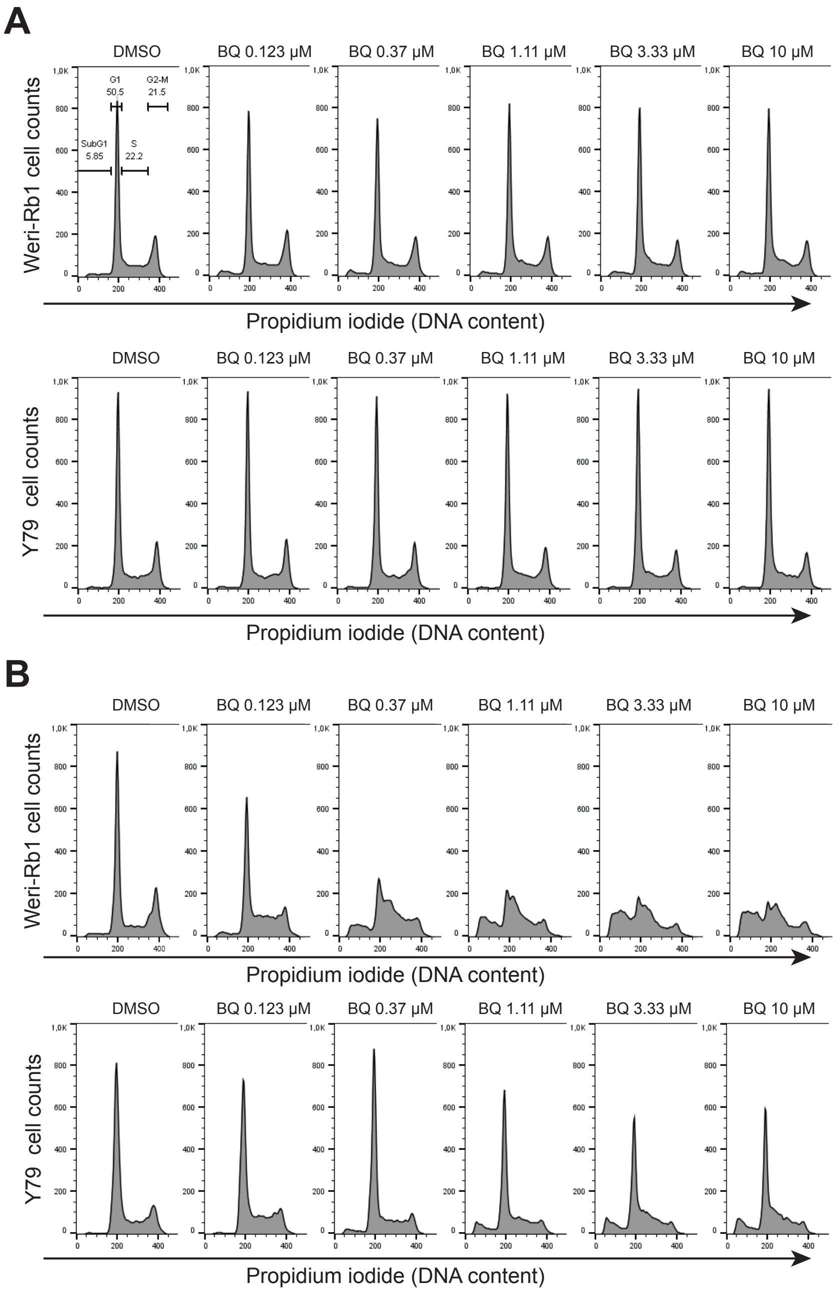

Supplementary Fig. 2. Cell cycle charts for Weri-Rb1 and Y79 cells treated with BQ from Fig. 2C and D. Propidium iodide flow cytometry was performed to obtain cell cycle distributions after treating the Weri-Rb1 and Y79 cells with BQ for 3 days (**A**) or 6 days (**B**) at the indicated concentrations.

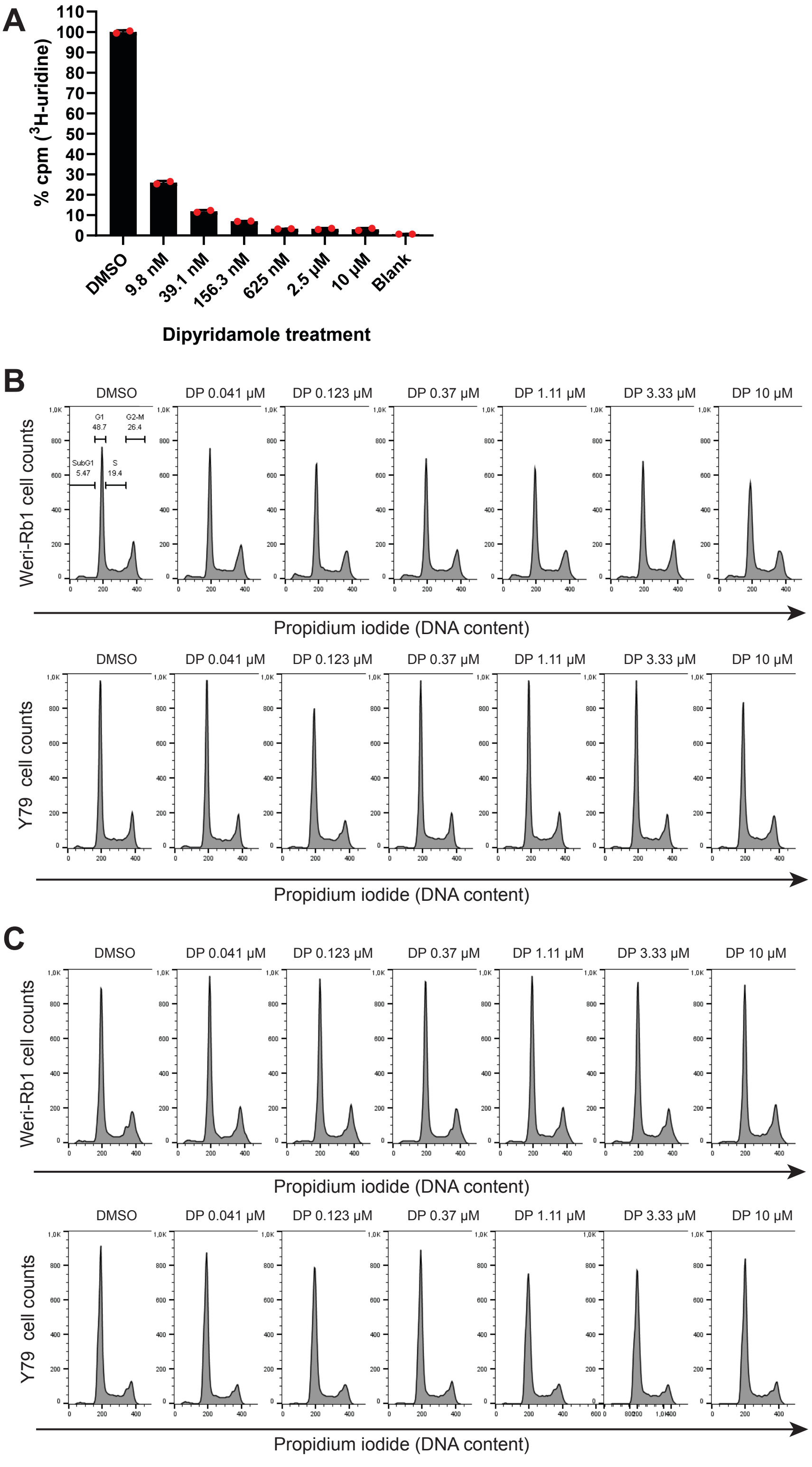

Supplementary Fig. 3. DP blocks uridine uptake and does not affect retinoblastoma cell growth. **A)** ARN8 melanoma cells were treated with DP as indicated for 15 min followed by a 60 sec ^{3}H-uridine pulse. After quick washes cells were harvested and treated with optiphase supermix. The mean counts per minute $\pm$ SD correspond to two technical replicates and are representative of 3 biological repeats. **B)** Propidium iodide flow cytometry was performed by treating the Weri-Rb1 cells and Y79 cells with DP for 3 days at the indicated concentrations. Data corresponds to bar graphs in Fig. 3C. **C)** Propidium iodide flow cytometry was performed by treating the Weri-Rb1 and Y79 cells with DP for 6 days at the indicated concentrations. Data corresponds to bar graphs in Fig. 3D.

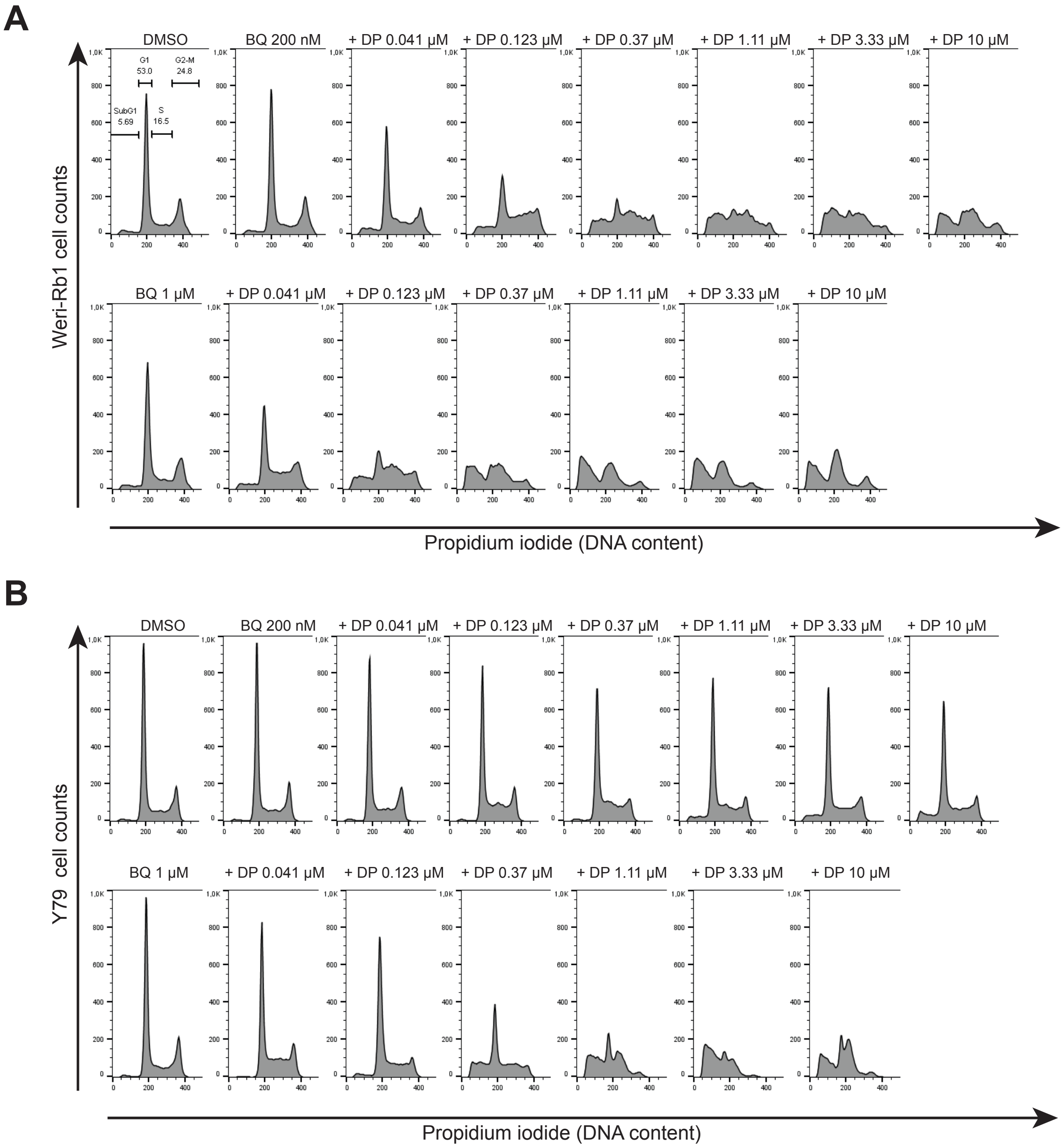

Supplementary Fig. 4. Cell cycle distribution data to find optimum concentration for DP in combination experiments. Propidium iodide flow cytometry was performed to obtain cell cycle distributions after treating Weri-Rb1 cells (**A**) or Y79 cells (**B**) for 3 days with BQ 200 nM or BQ 1 µM alone or in combination with increasing concentrations of DP (+ DP).

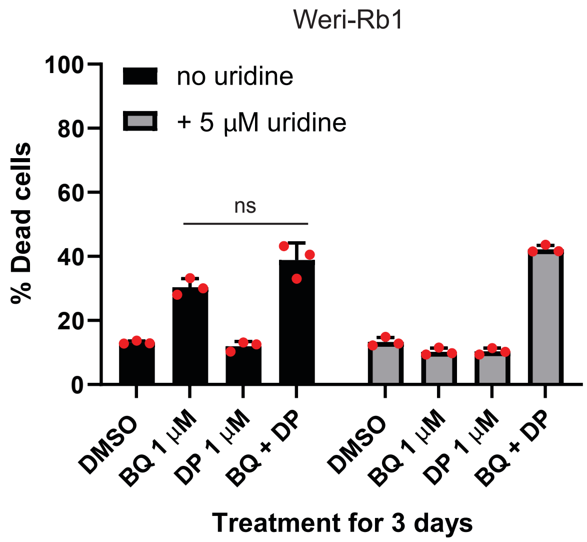

Supplementary Fig. 5. Confirmation that DP inhibits uridine uptake in combination treatments. Weri-Rb1 cells were treated as indicated in serum replacement 3 (instead of fetal bovine serum) in the presence or absence of 5 µM uridine. Propidium iodide flow cytometry was performed to determine the percentage of cells with a <2N DNA content. Data is given as mean $\pm$ SD for three technical replicates and are representative of 3 biological repeats. Student's t-test was performed to compare between BQ and compound combination treated group in absence of uridine; $p < 0.05$ was considered as significant, ns (not significant).

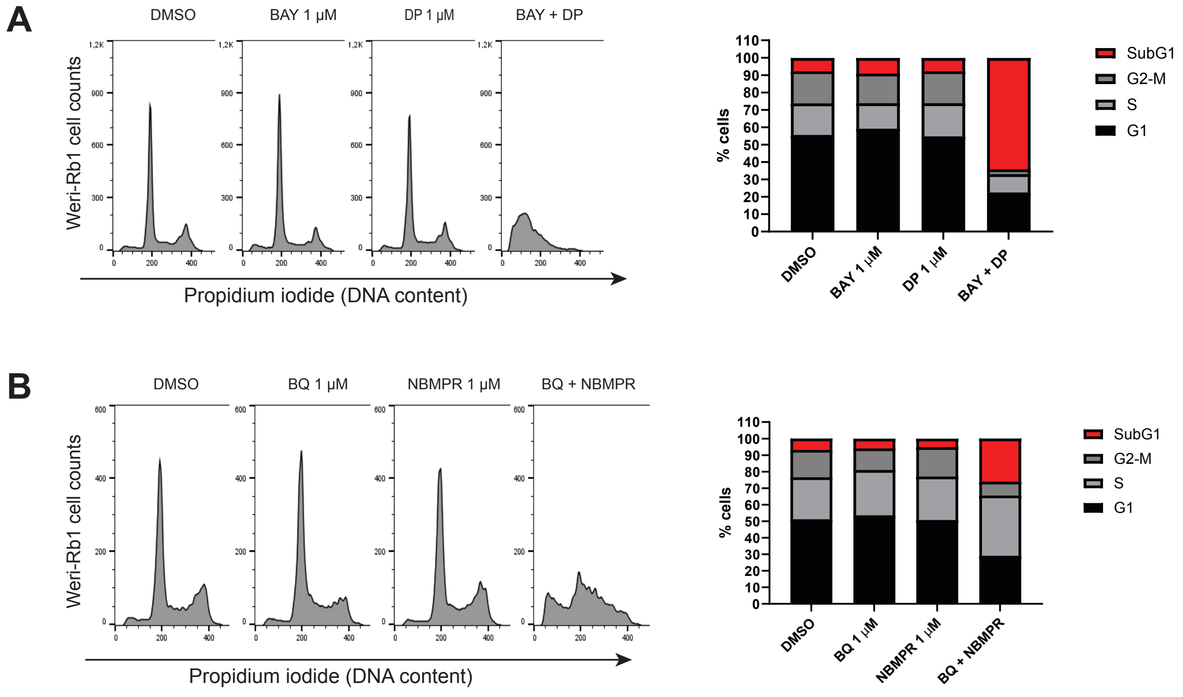

Supplementary Fig. 6. Other DHODH and nucleoside transport inhibitors elicit similar responses in retinoblastoma cells. **A)** Propidium iodide flow cytometry was performed after treating Weri-Rb1 cells with DMSO, BAY2402234, DP or the combination for 3 days. **B)** Propidium iodide flow cytometry was performed after treating Weri-Rb1 cells with DMSO, BQ, NBMPR or the combination for 3 days. Cell cycle profiles and corresponding bar graphs are shown.

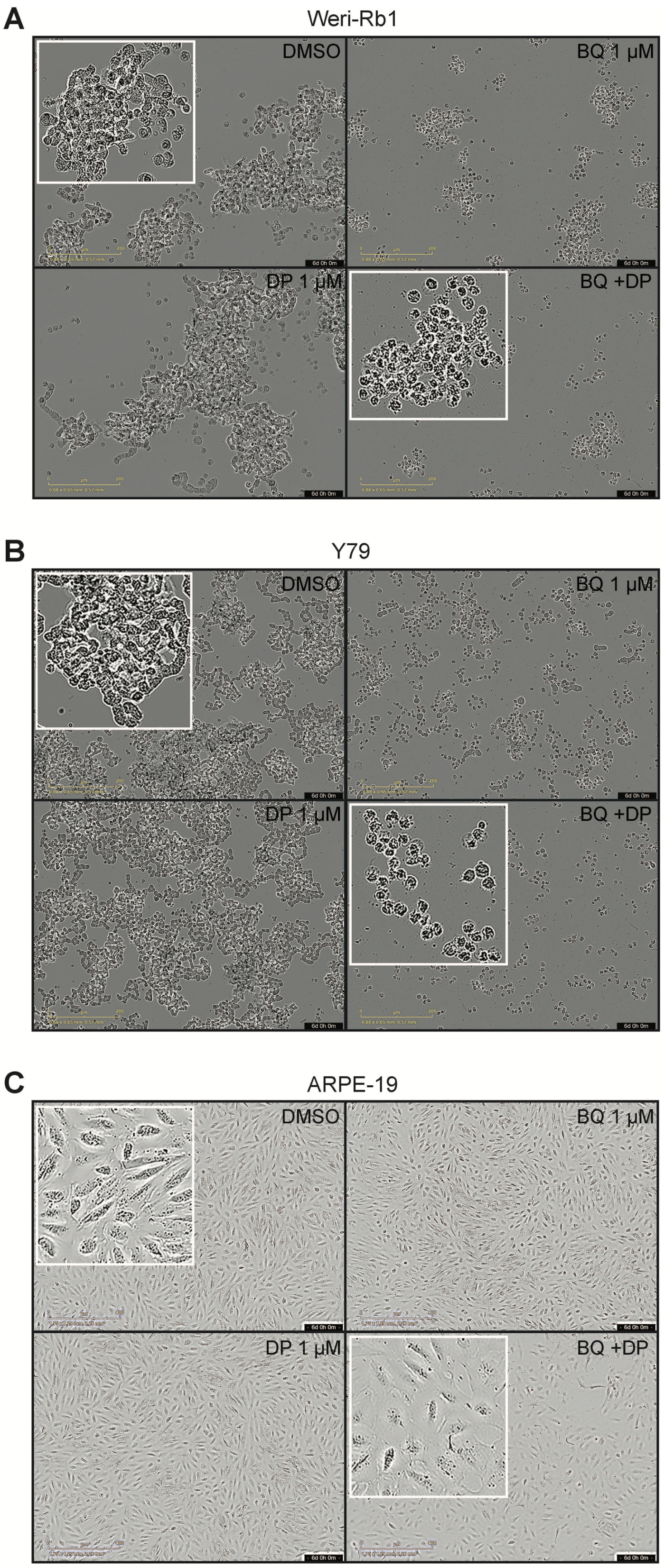

Supplementary Fig. 7. Live cell imaging of retinoblastoma and ARPE-19 cells after compound treatment for 6 days. **A-B** Weri-Rb1 or Y79 cells were treated as indicated for 6 days. Images were taken every 4 h at 20x magnification using the phase contrast feature of IncuCyte S3 system and video files were made to see morphological changes over time. Images taken at the 6-day time point are shown here. **C)** ARPE-19 cells were treated as indicated for 6 days with images taken every 4 h at 10x magnification. Here images taken at the 6-day time point are shown. Inserts show magnified images of the cells.

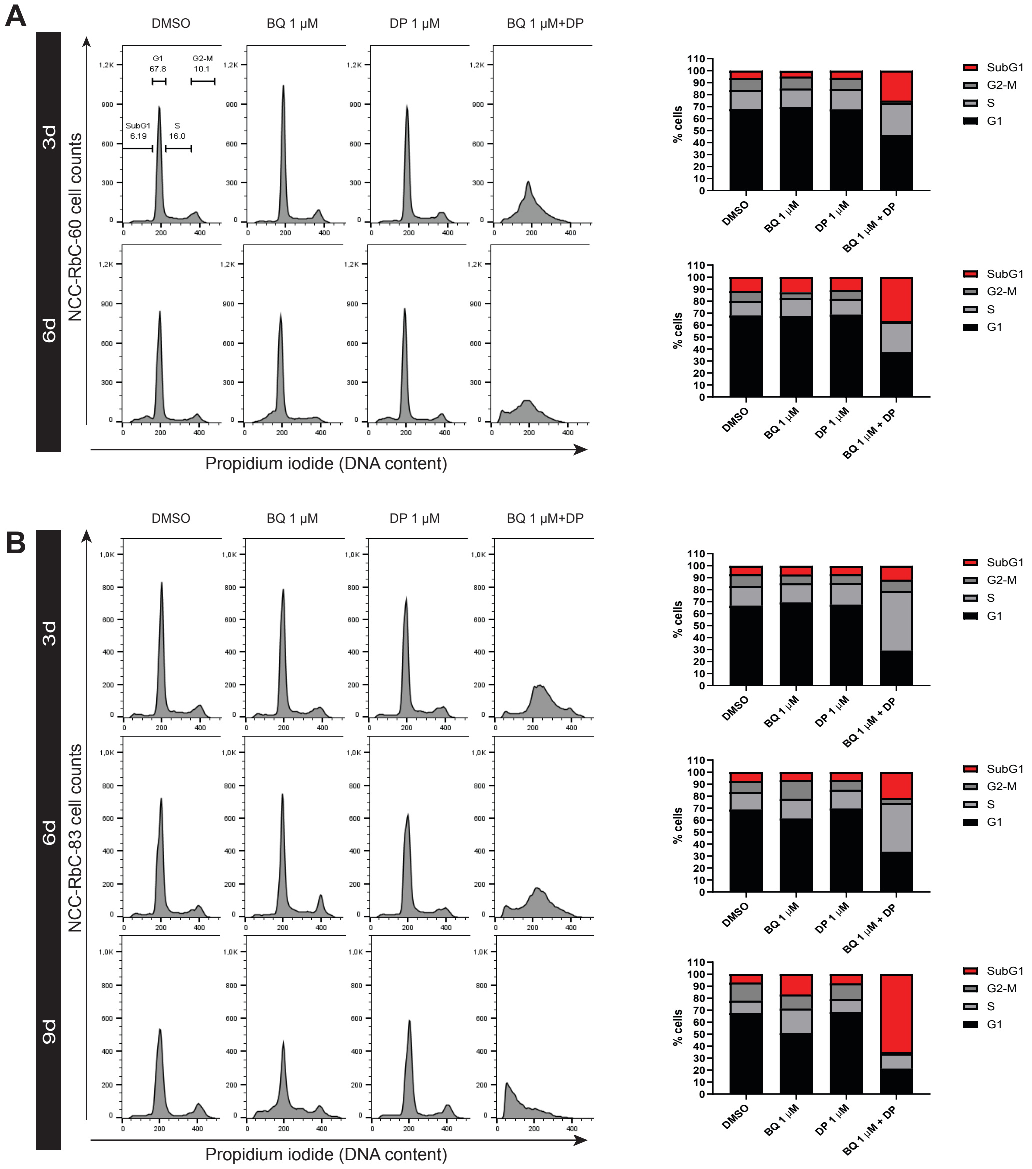

Supplementary Fig. 8. Effects of DHODHi in combination with dipyridamole on other retinoblastoma cell lines. Propidium iodide flow cytometry was performed after treating NCC-RbC-60 (**A**) and NCC-RbC-83 (**B**) cells as indicated. The cell cycle distribution after each treatment is shown on the left, and the % of cells in each cell cycle phase is represented by a bar graph on the right.

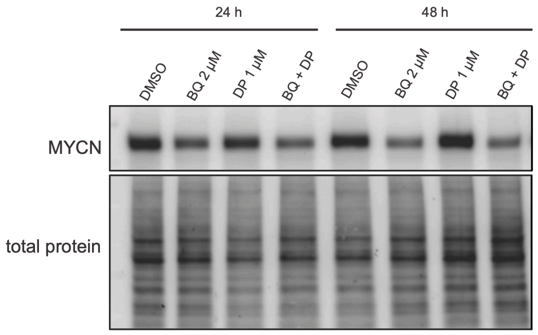

Supplementary Fig. 9. Effect brequinar and dipyridamole on MYCN protein levels. MYCN levels in Y79 cells upon the indicated treatments.

Whole western blots (1)

Figure 2A

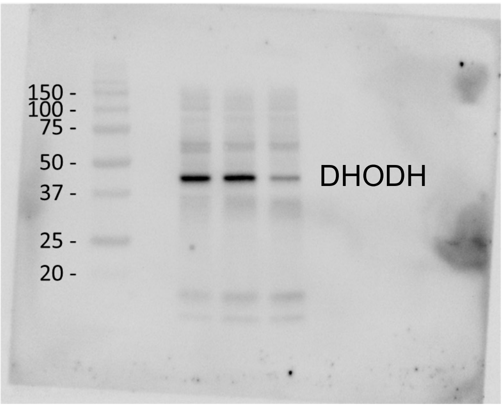

Total protein

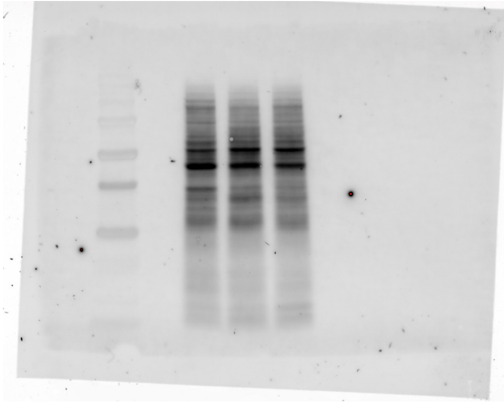

Figure 6C

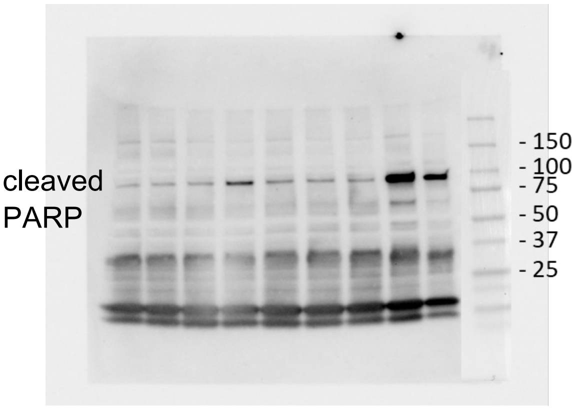

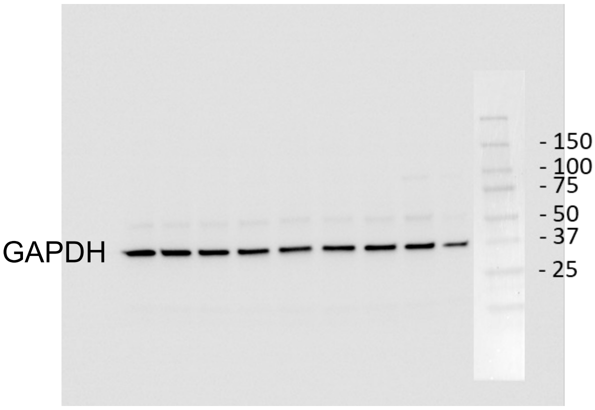

Whole western blots (2)

Figure 7A (top panels)

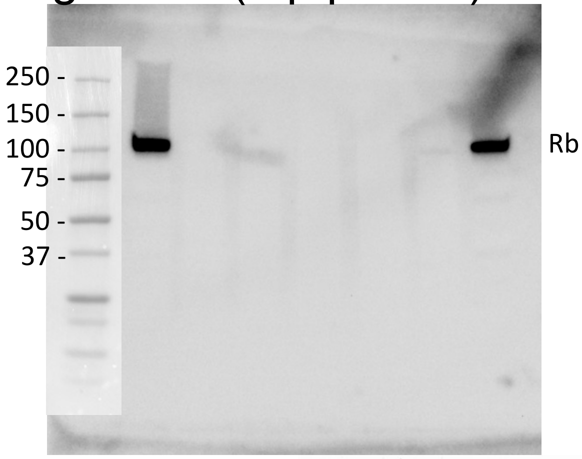

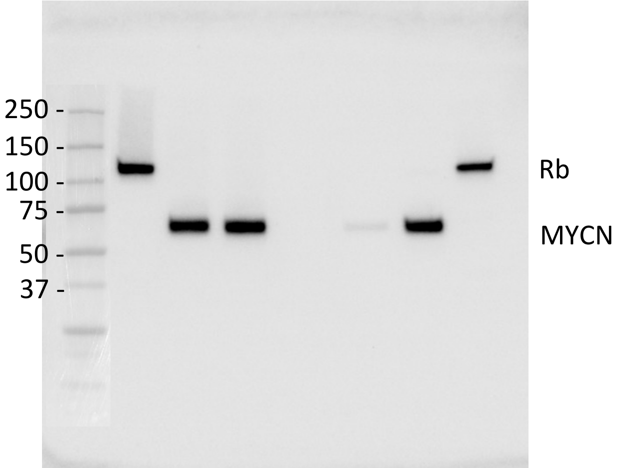

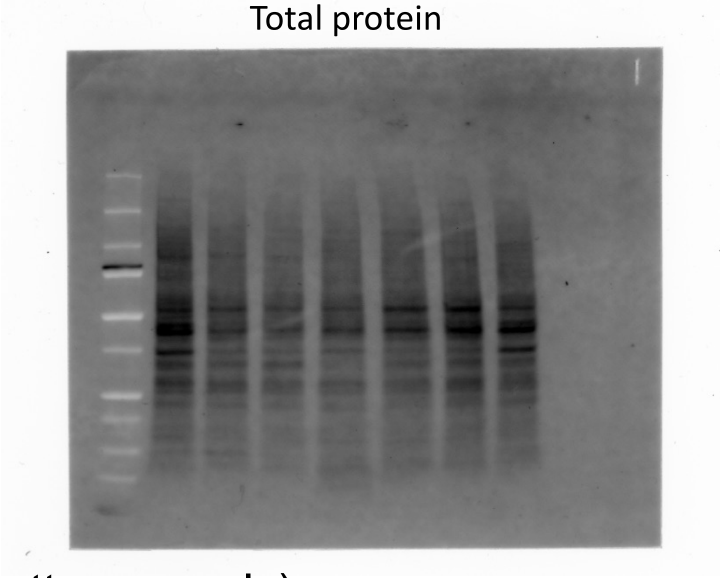

Figure 7A (bottom panels)

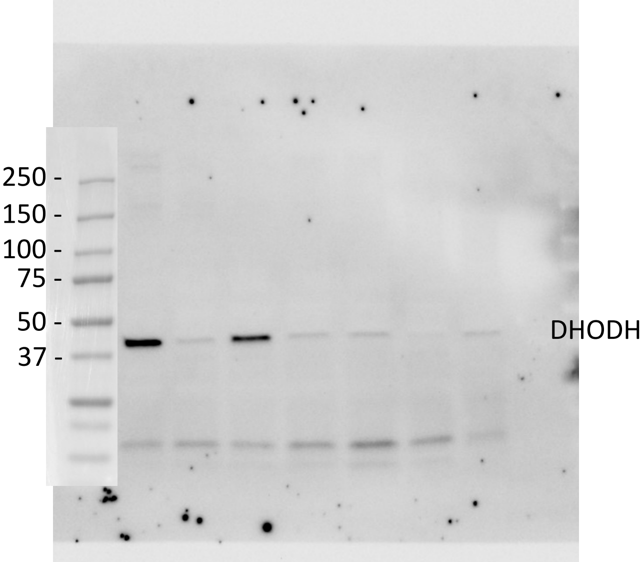

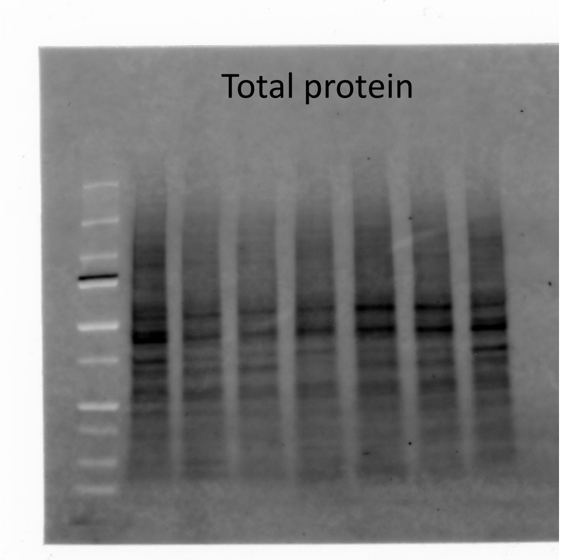

Whole western blots (3)

Figure 7B

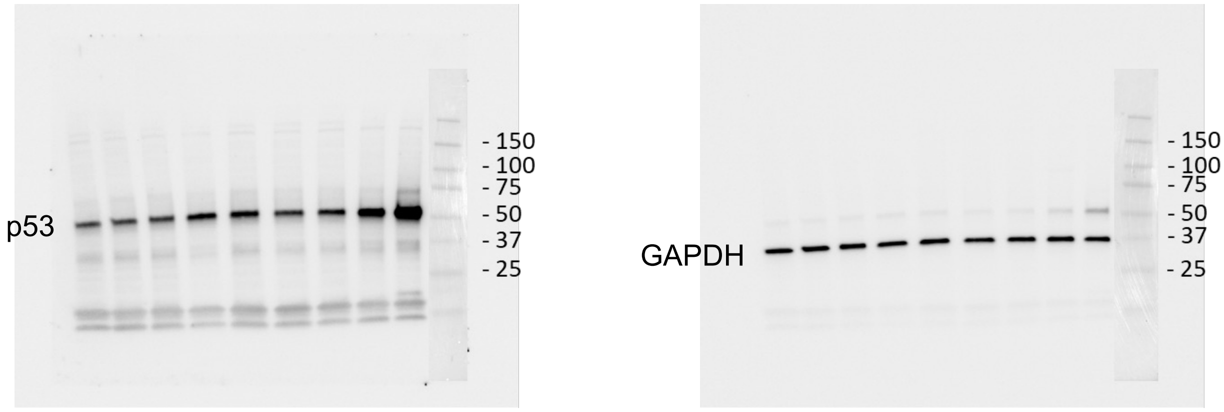

Figure 7C

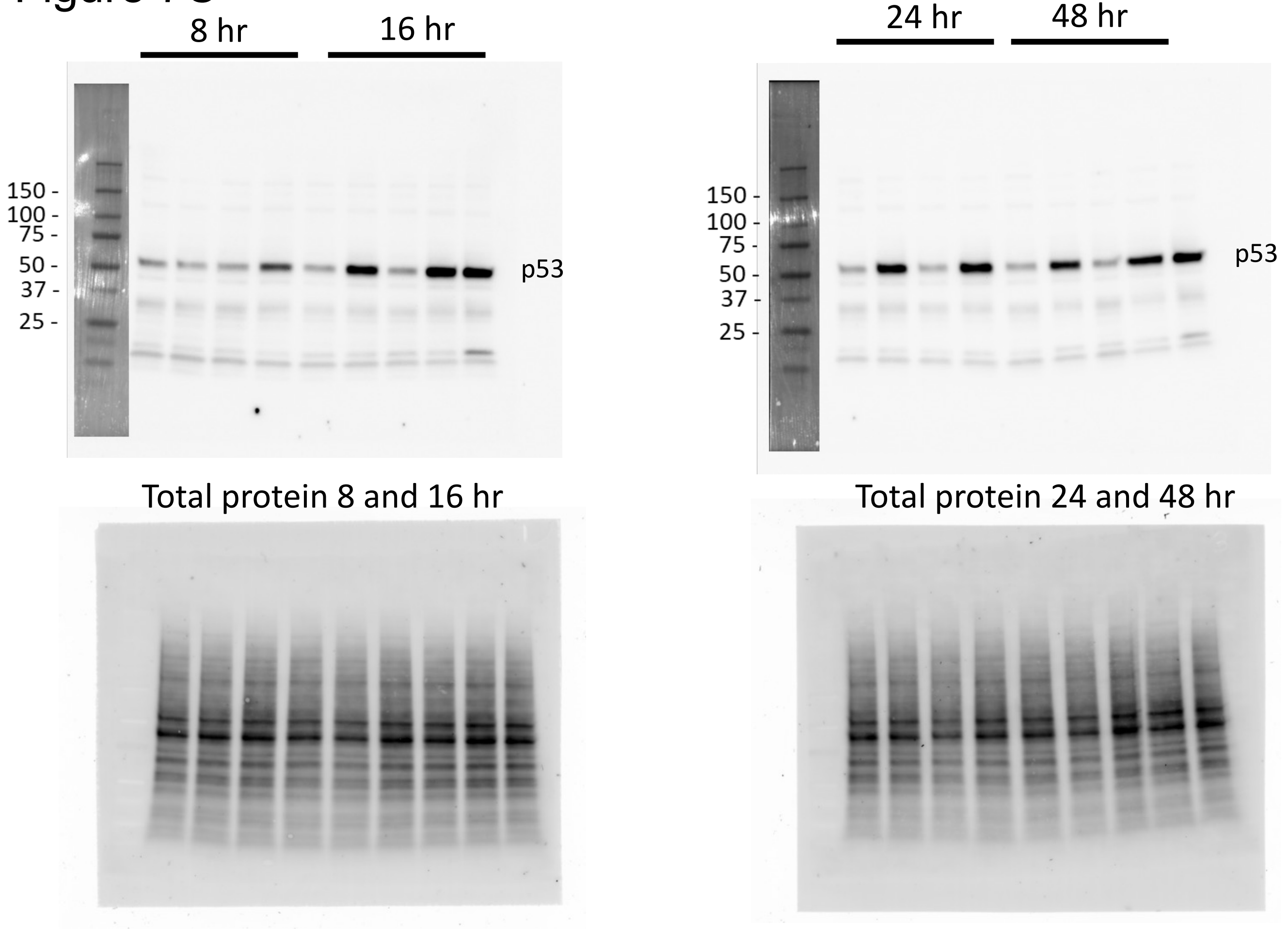

Whole western blots (4)

Figure S9

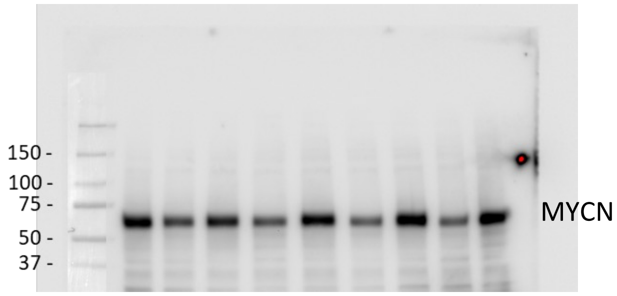

Total protein

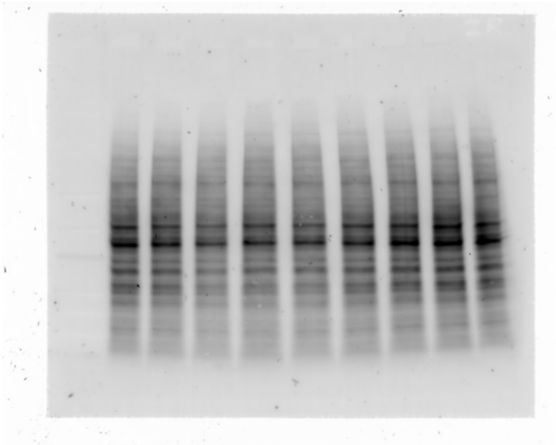

Supplement: Multimedia component 1 [file mmc1.pdf]
